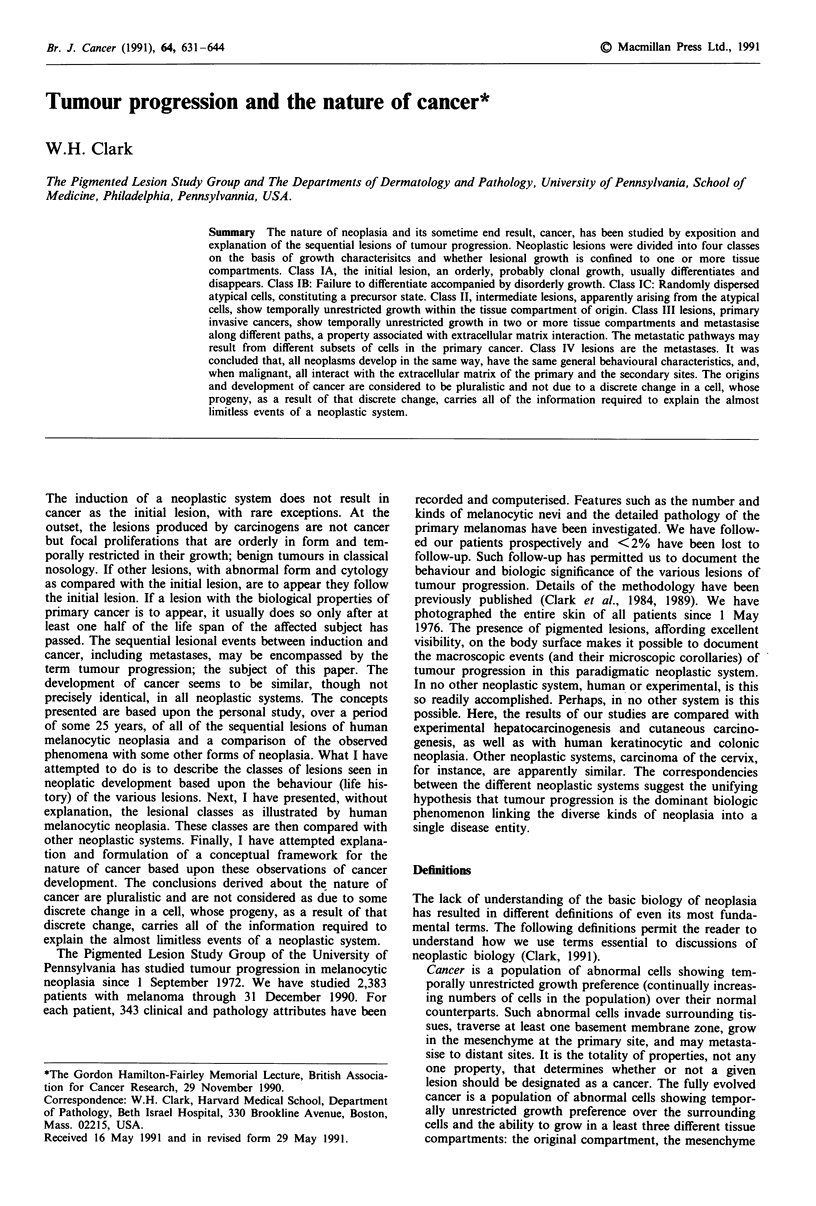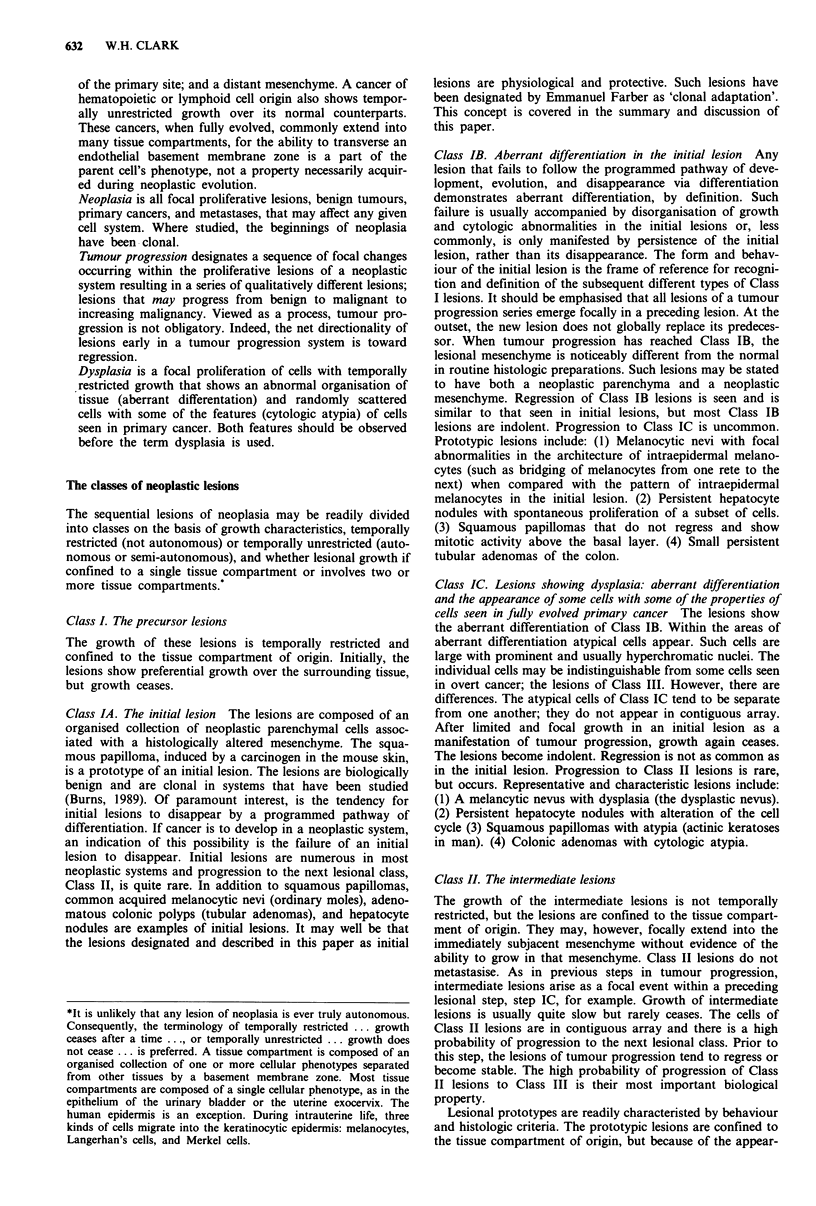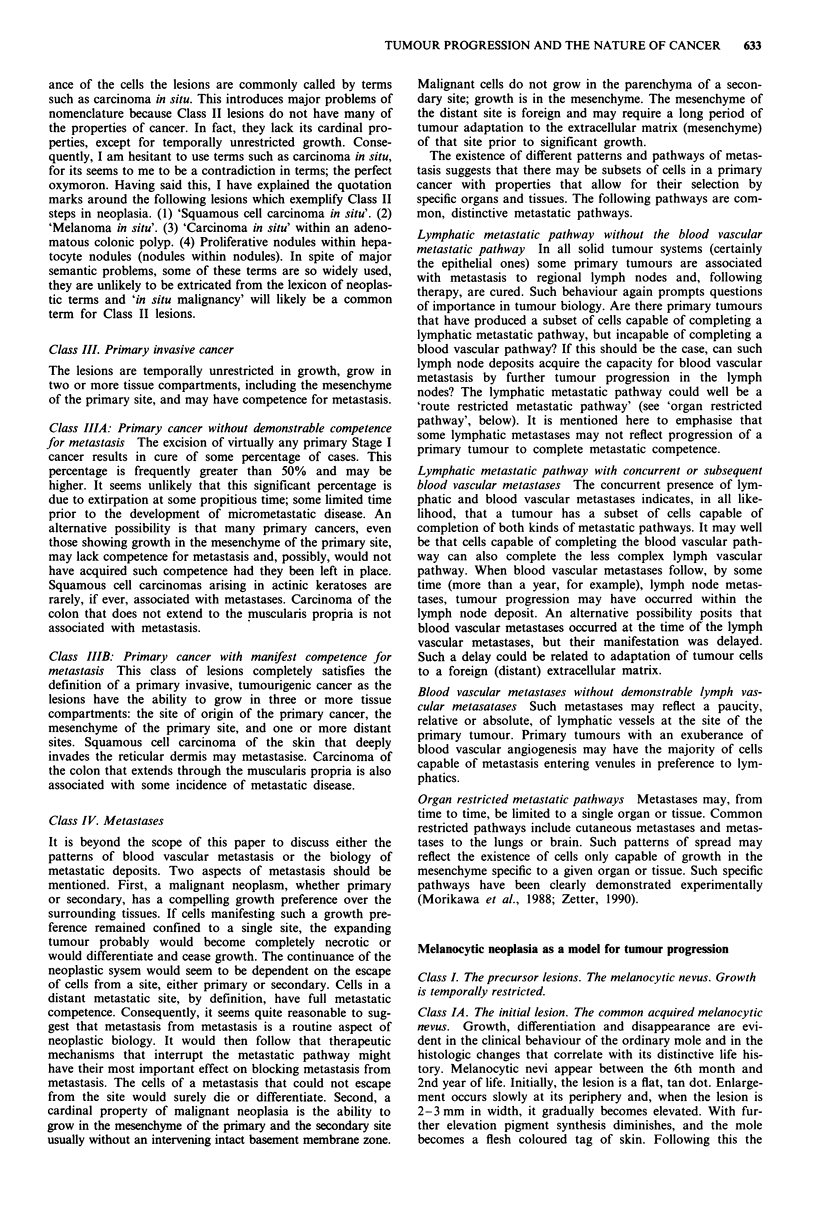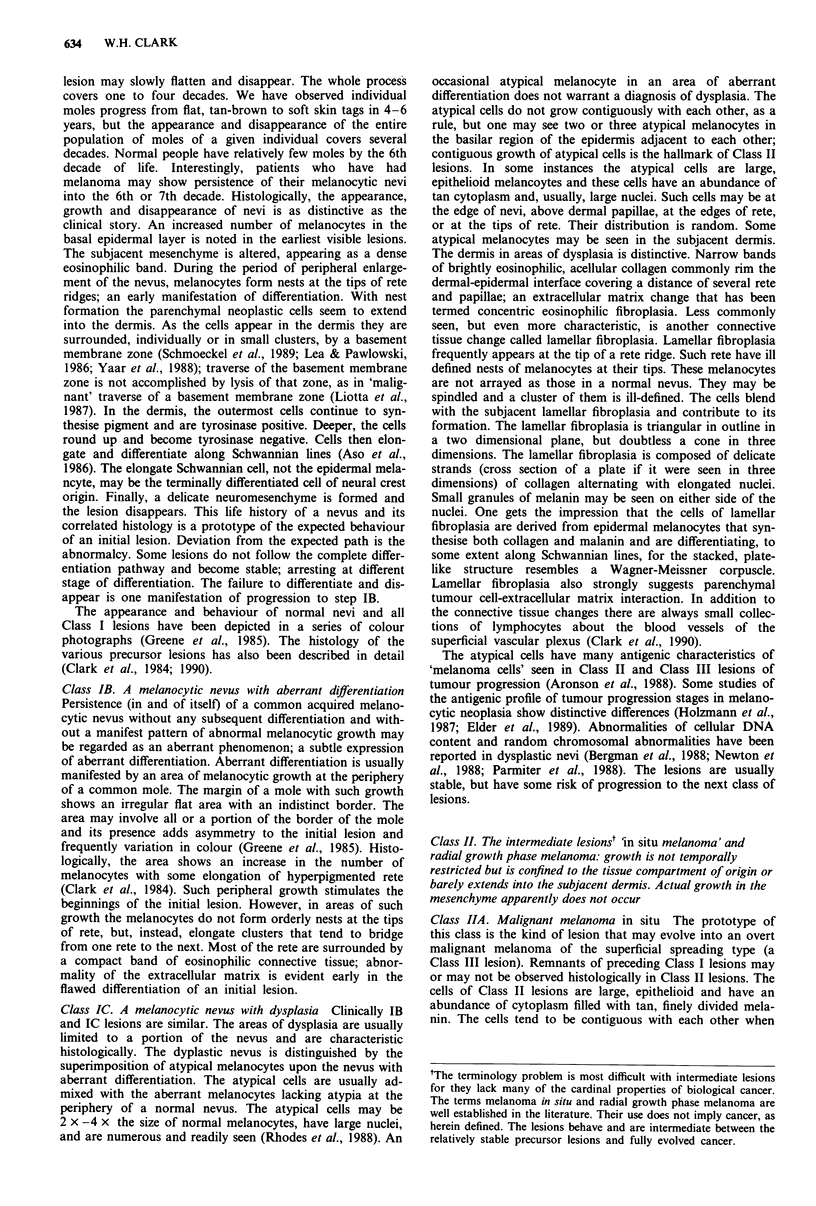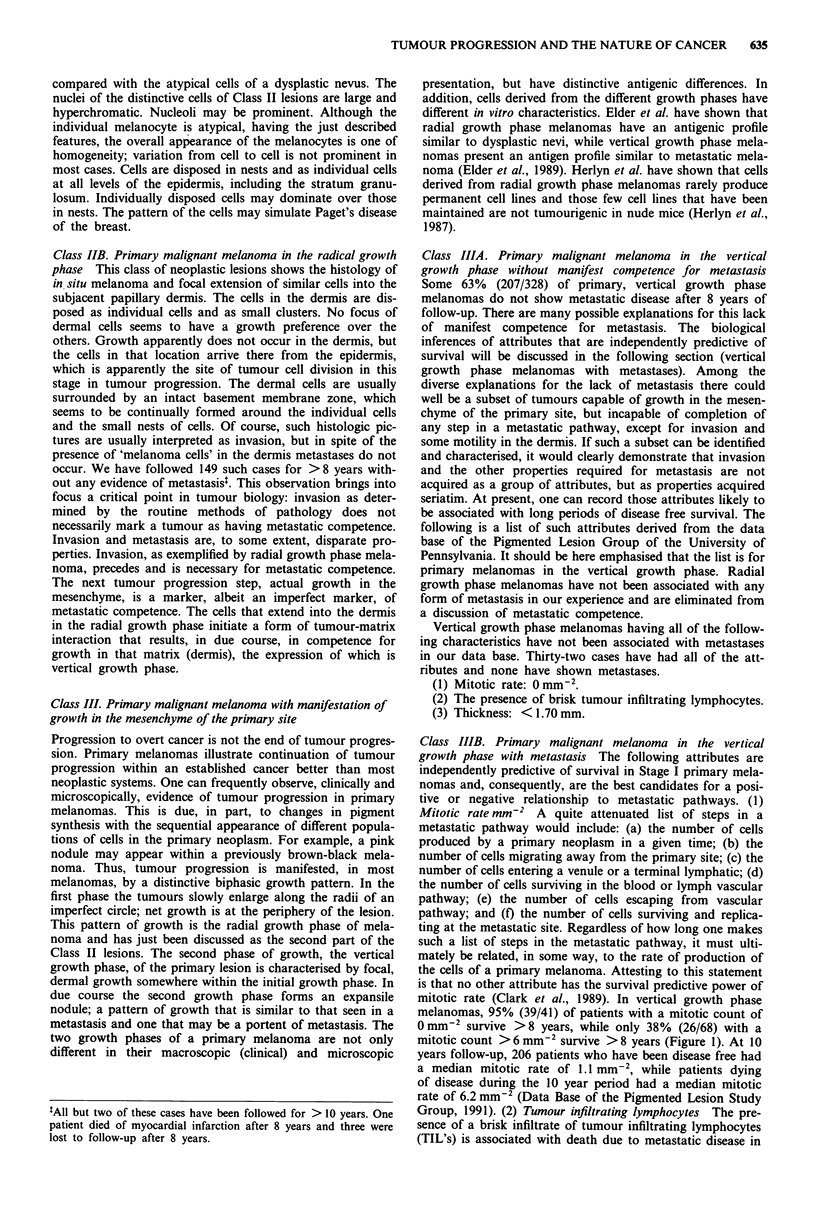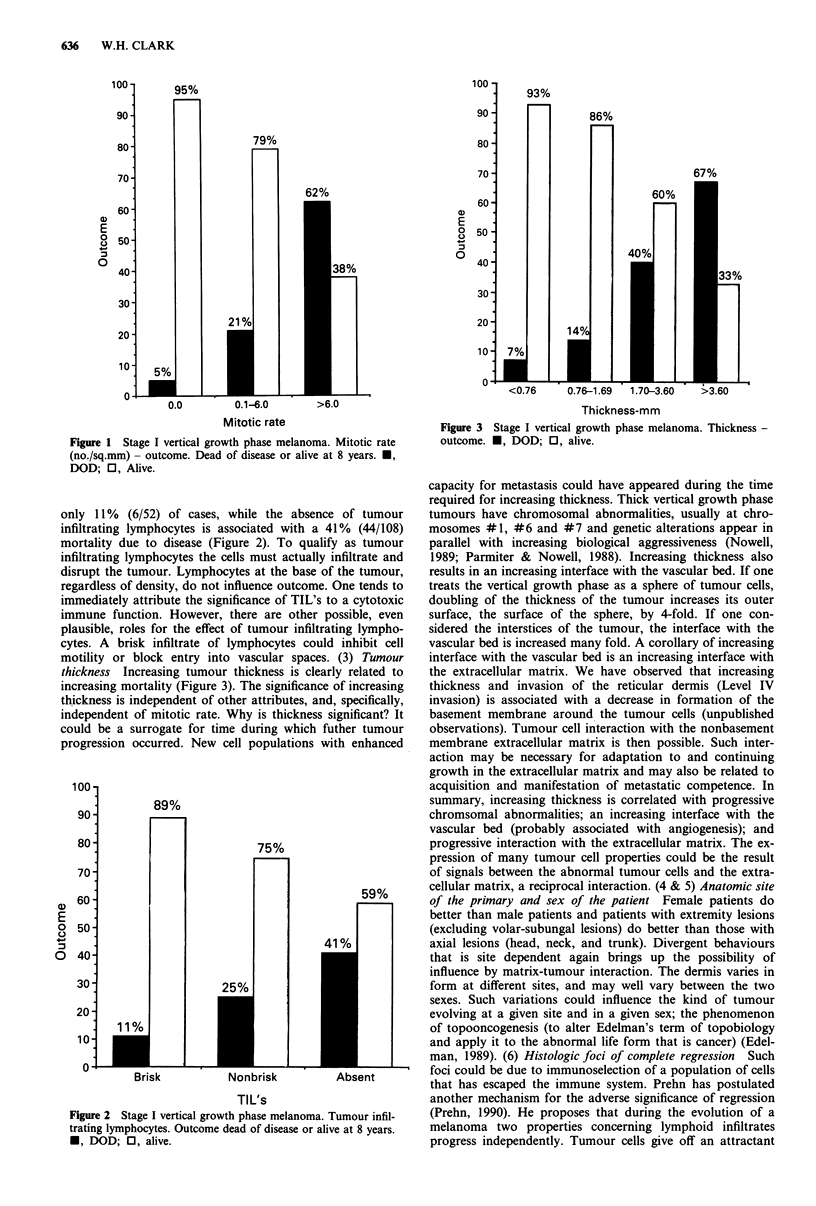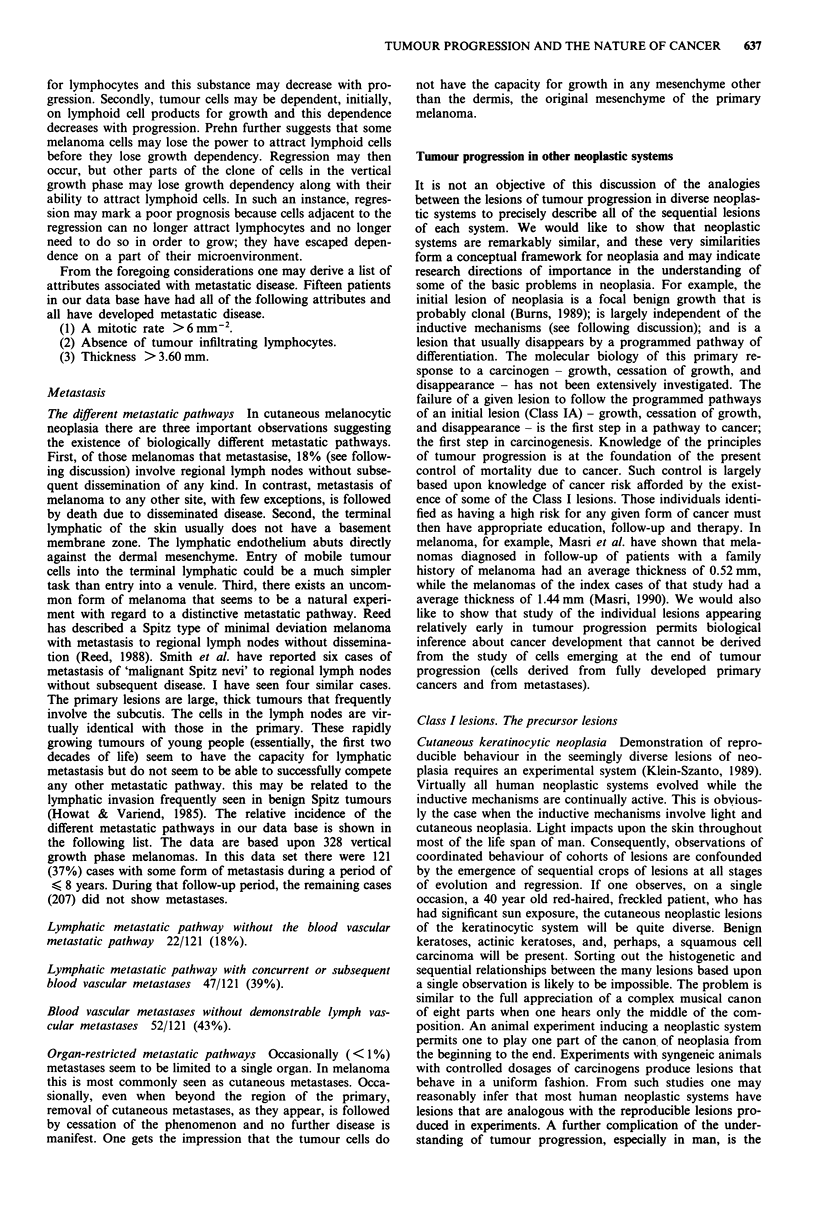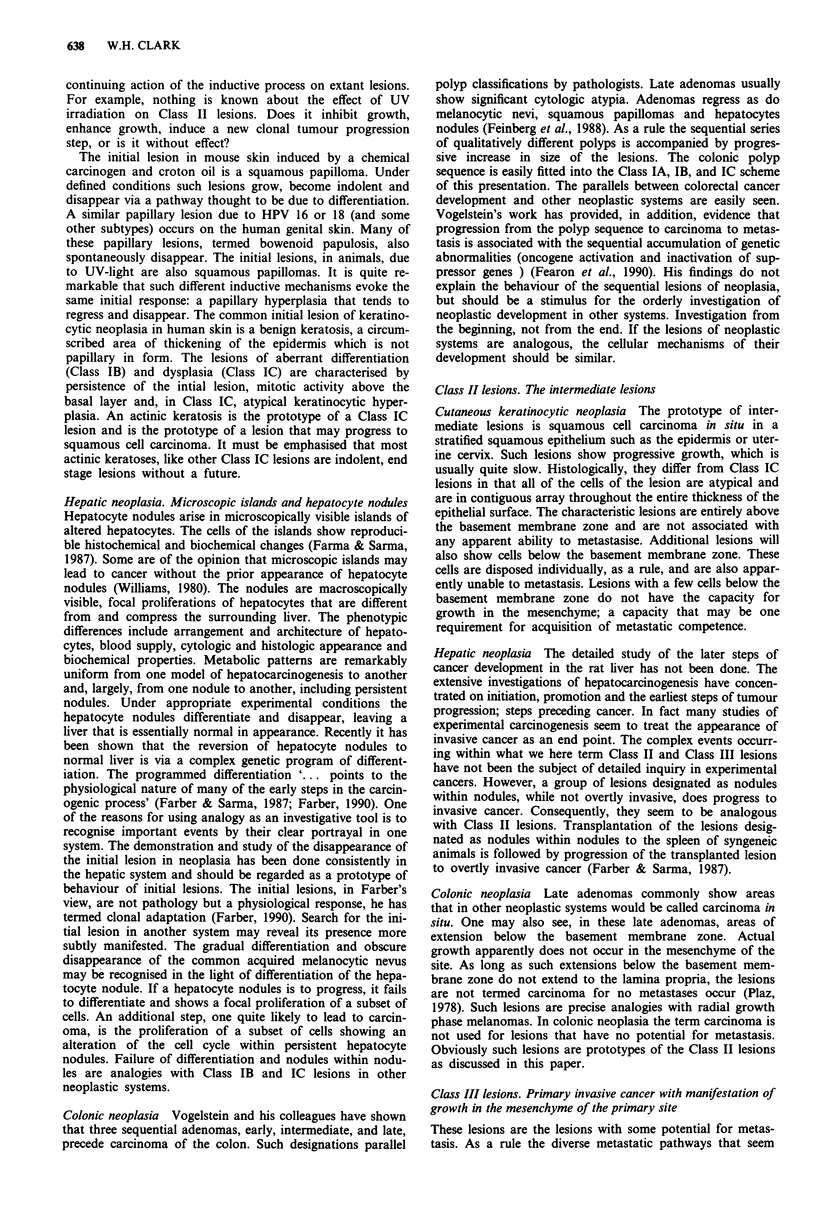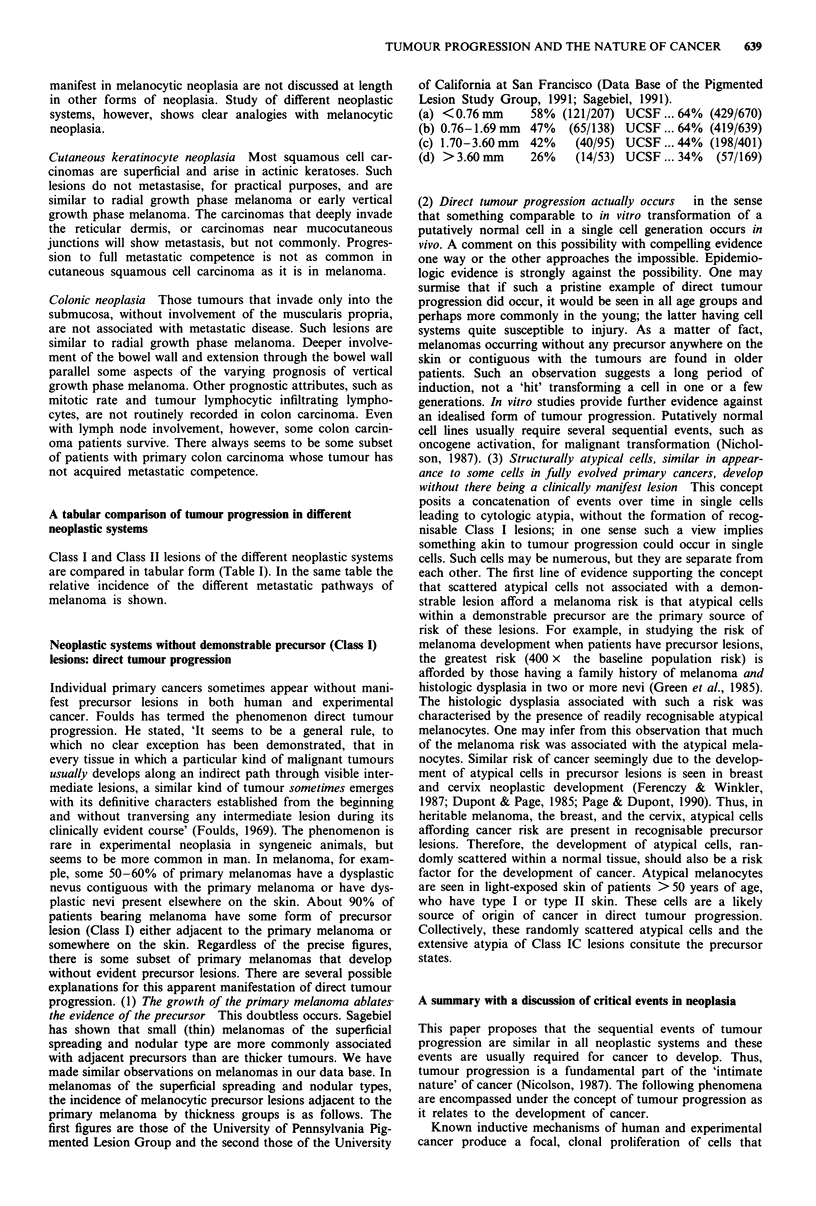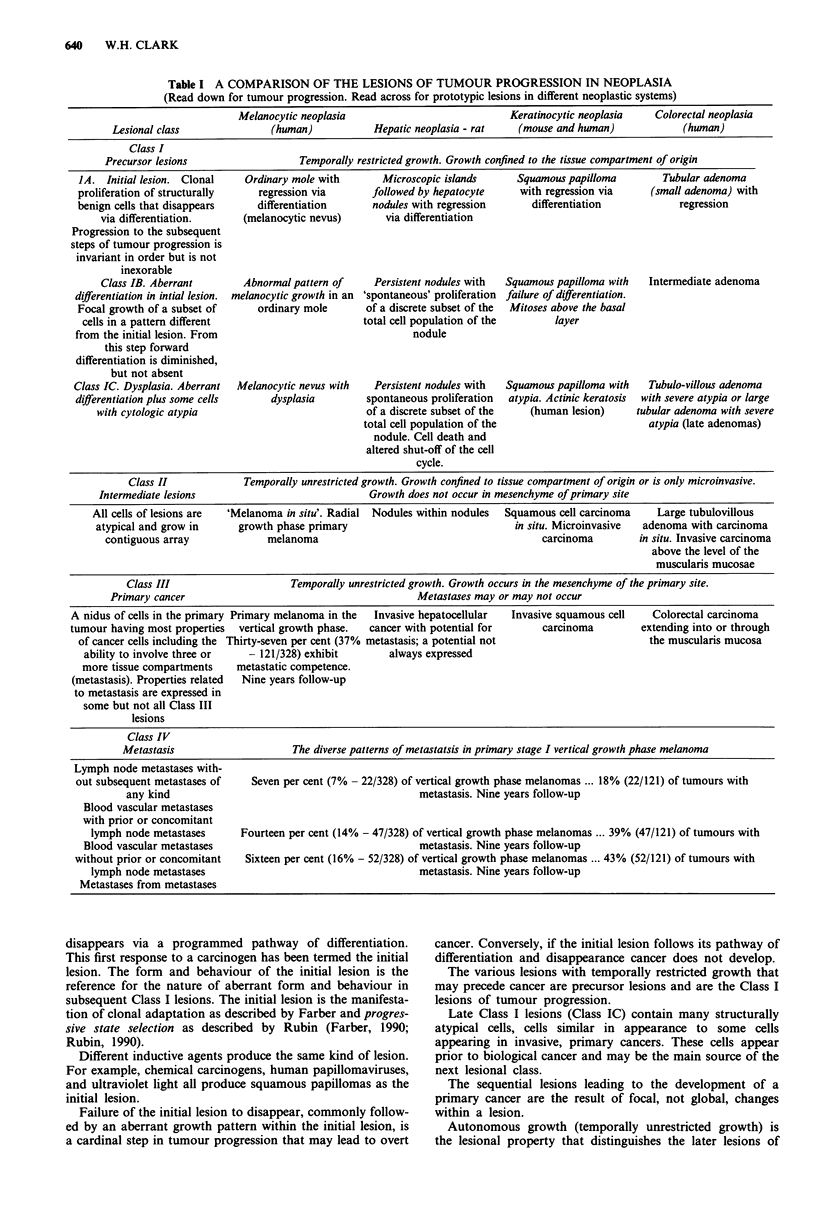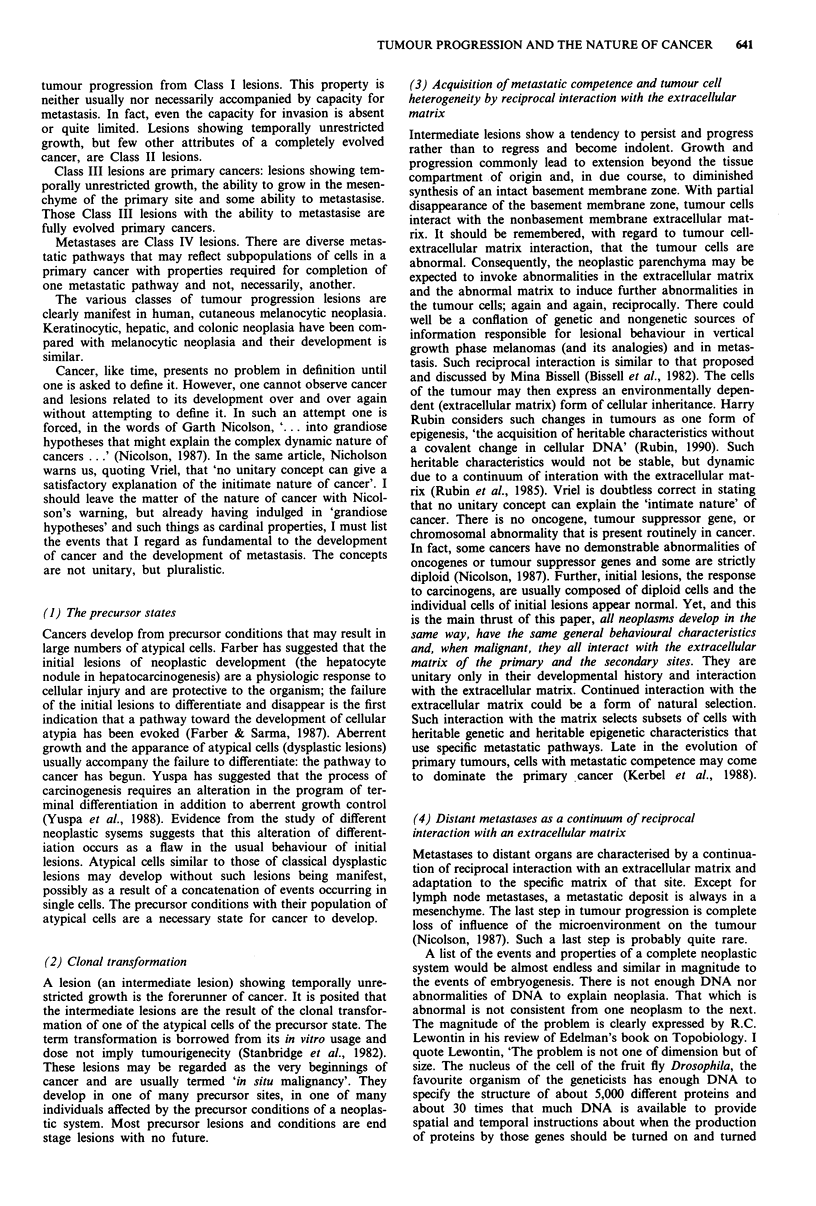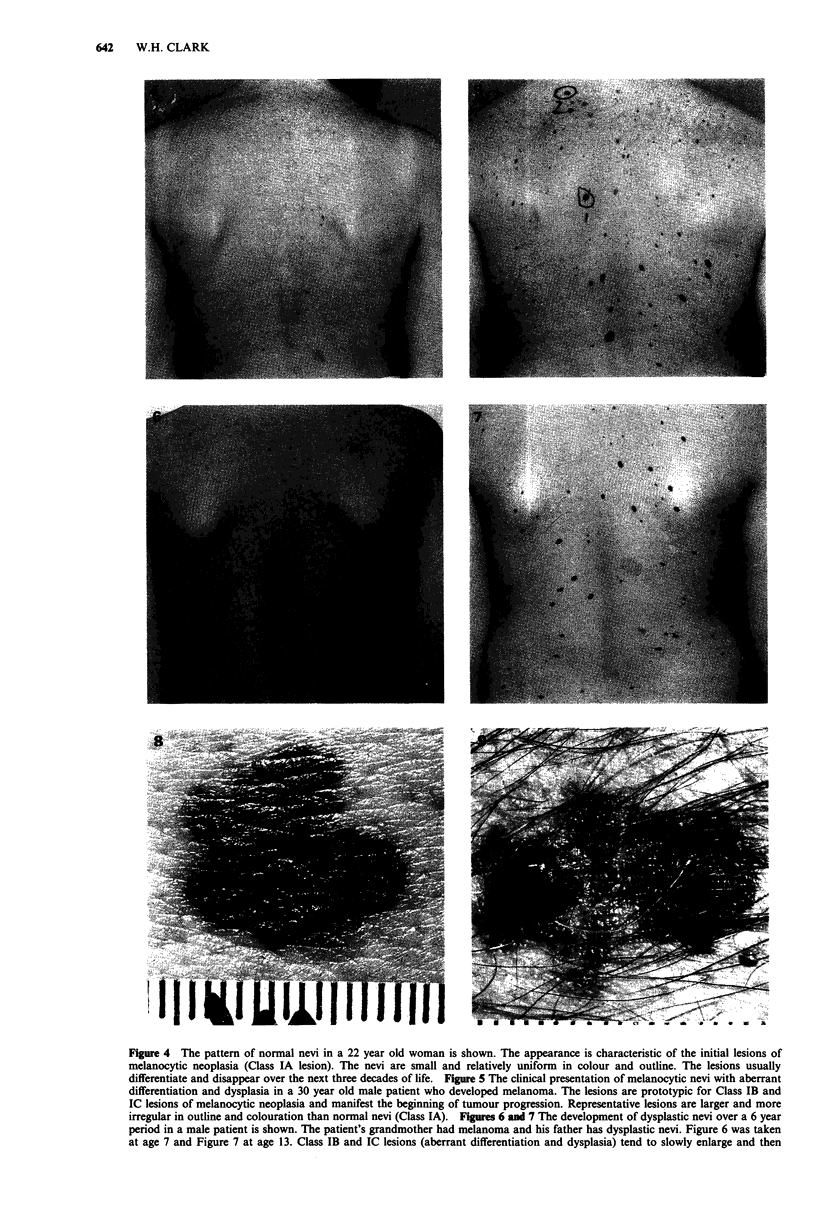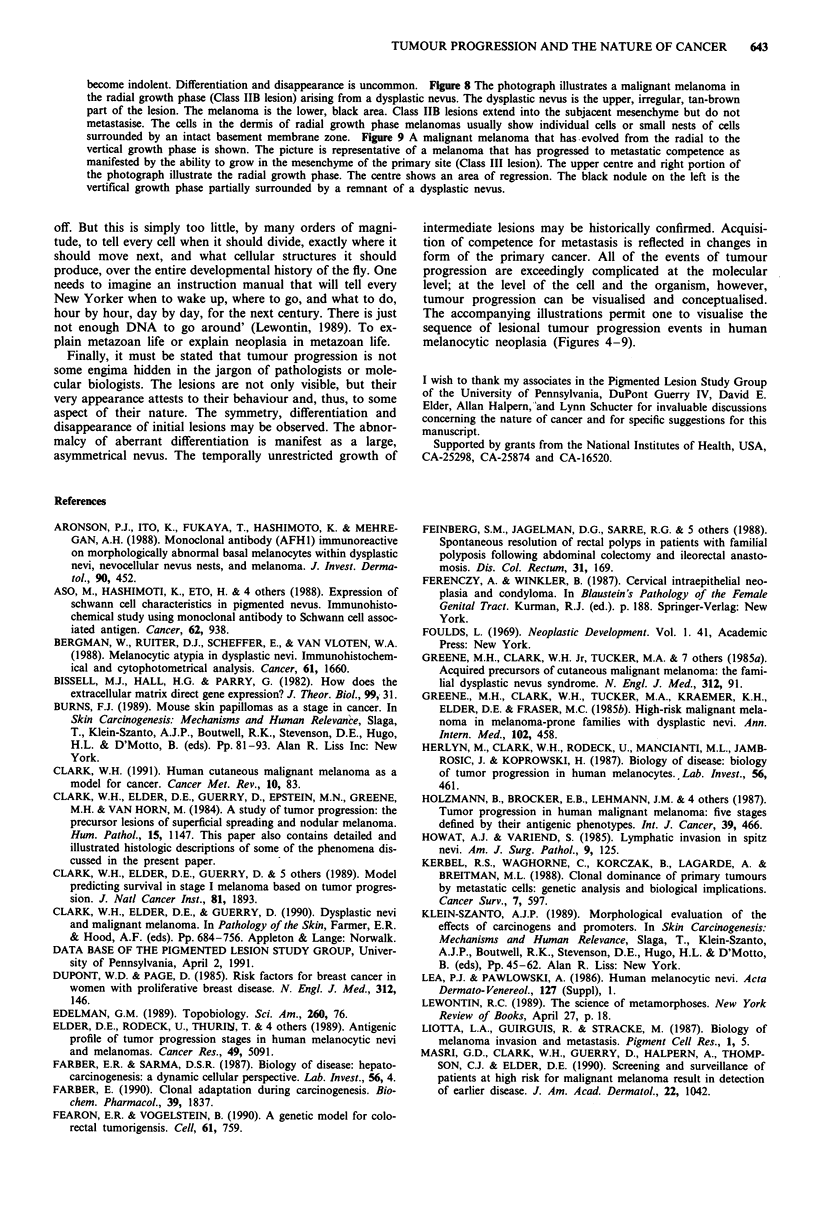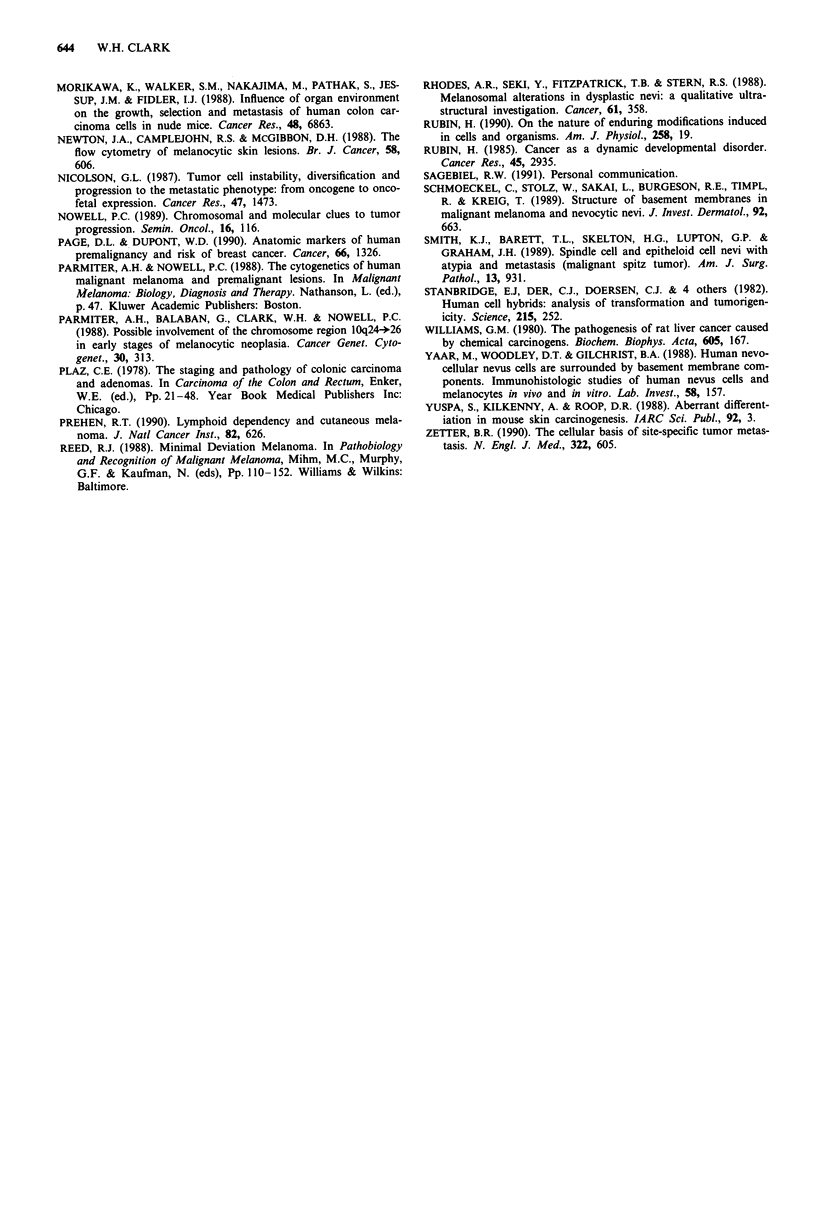# Tumour progression and the nature of cancer.

**DOI:** 10.1038/bjc.1991.375

**Published:** 1991-10

**Authors:** W. H. Clark

**Affiliations:** Pigmented Lesion Study Group, University of Pennsylvania, School of Medicine, Philadelphia.

## Abstract

**Images:**


Br. J. Cancer (1991), 64, 631–644 © Macmillan Press Ltd., 1991

### Tumour progression and the nature of cancer*

W.H. Clark

*The Pigmented Lesion Study Group and The Departments of Dermatology and Pathology, University of Pennsylvania, School of Medicine, Philadelphia, Pennsylvannia, USA.*

**Summary** The nature of neoplasia and its sometime end result, cancer, has been studied by exposition and explanation of the sequential lesions of tumour progression. Neoplastic lesions were divided into four classes on the basis of growth characterisitcs and whether lesional growth is confined to one or more tissue compartments. Class IA, the initial lesion, an orderly, probably clonal growth, usually differentiates and disappears. Class IB: Failure to differentiate accompanied by disorderly growth. Class IC: Randomly dispersed atypical cells, constituting a precursor state. Class II, intermediate lesions, apparently arising from the atypical cells, show temporally unrestricted growth within the tissue compartment of origin. Class III lesions, primary invasive cancers, show temporally unrestricted growth in two or more tissue compartments and metastasise along different paths, a property associated with extracellular matrix interaction. The metastatic pathways may result from different subsets of cells in the primary cancer. Class IV lesions are the metastases. It was concluded that, all neoplasms develop in the same way, have the same general behavioural characteristics, and, when malignant, all interact with the extracellular matrix of the primary and the secondary sites. The origins and development of cancer are considered to be pluralistic and not due to a discrete change in a cell, whose progeny, as a result of that discrete change, carries all of the information required to explain the almost limitless events of a neoplastic system.

The induction of a neoplastic system does not result in cancer as the initial lesion, with rare exceptions. At the outset, the lesions produced by carcinogens are not cancer but focal proliferations that are orderly in form and temporally restricted in their growth; benign tumours in classical nosology. If other lesions, with abnormal form and cytology as compared with the initial lesion, are to appear they follow the initial lesion. If a lesion with the biological properties of primary cancer is to appear, it usually does so only after at least one half of the life span of the affected subject has passed. The sequential lesional events between induction and cancer, including metastases, may be encompassed by the term tumour progression; the subject of this paper. The development of cancer seems to be similar, though not precisely identical, in all neoplastic systems. The concepts presented are based upon the personal study, over a period of some 25 years, of all of the sequential lesions of human melanocytic neoplasia and a comparison of the observed phenomena with some other forms of neoplasia. What I have attempted to do is to describe the classes of lesions seen in neoplatic development based upon the behaviour (life history) of the various lesions. Next, I have presented, without explanation, the lesional classes as illustrated by human melanocytic neoplasia. These classes are then compared with other neoplastic systems. Finally, I have attempted explanation and formulation of a conceptual framework for the nature of cancer based upon these observations of cancer development. The conclusions derived about the nature of cancer are pluralistic and are not considered as due to some discrete change in a cell, whose progeny, as a result of that discrete change, carries all of the information required to explain the almost limitless events of a neoplastic system.

The Pigmented Lesion Study Group of the University of Pennsylvania has studied tumour progression in melanocytic neoplasia since 1 September 1972. We have studied 2,383 patients with melanoma through 31 December 1990. For each patient, 343 clinical and pathology attributes have been recorded and computerised. Features such as the number and kinds of melanocytic nevi and the detailed pathology of the primary melanomas have been investigated. We have followed our patients prospectively and $<2\%$ have been lost to follow-up. Such follow-up has permitted us to document the behaviour and biologic significance of the various lesions of tumour progression. Details of the methodology have been previously published (Clark *et al.*, 1984, 1989). We have photographed the entire skin of all patients since 1 May 1976. The presence of pigmented lesions, affording excellent visibility, on the body surface makes it possible to document the macroscopic events (and their microscopic corollaries) of tumour progression in this paradigmatic neoplastic system. In no other neoplastic system, human or experimental, is this so readily accomplished. Perhaps, in no other system is this possible. Here, the results of our studies are compared with experimental hepatocarcinogenesis and cutaneous carcinogenesis, as well as with human keratinocytic and colonic neoplasia. Other neoplastic systems, carcinoma of the cervix, for instance, are apparently similar. The correspondencies between the different neoplastic systems suggest the unifying hypothesis that tumour progression is the dominant biologic phenomenon linking the diverse kinds of neoplasia into a single disease entity.

### Definitions

The lack of understanding of the basic biology of neoplasia has resulted in different definitions of even its most fundamental terms. The following definitions permit the reader to understand how we use terms essential to discussions of neoplastic biology (Clark, 1991).

*Cancer* is a population of abnormal cells showing temporally unrestricted growth preference (continually increasing numbers of cells in the population) over their normal counterparts. Such abnormal cells invade surrounding tissues, traverse at least one basement membrane zone, grow in the mesenchyme at the primary site, and may metastasise to distant sites. It is the totality of properties, not any one property, that determines whether or not a given lesion should be designated as a cancer. The fully evolved cancer is a population of abnormal cells showing temporally unrestricted growth preference over the surrounding cells and the ability to grow in a least three different tissue compartments: the original compartment, the mesenchyme

*The Gordon Hamilton-Fairley Memorial Lecture, British Association for Cancer Research, 29 November 1990.

Correspondence: W.H. Clark, Harvard Medical School, Department of Pathology, Beth Israel Hospital, 330 Brookline Avenue, Boston, Mass. 02215, USA.

Received 16 May 1991 and in revised form 29 May 1991.

of the primary site; and a distant mesenchyme. A cancer of hematopoietic or lymphoid cell origin also shows temporally unrestricted growth over its normal counterparts. These cancers, when fully evolved, commonly extend into many tissue compartments, for the ability to transverse an endothelial basement membrane zone is a part of the parent cell's phenotype, not a property necessarily acquired during neoplastic evolution.

*Neoplasia* is all focal proliferative lesions, benign tumours, primary cancers, and metastases, that may affect any given cell system. Where studied, the beginnings of neoplasia have been clonal.

*Tumour progression* designates a sequence of focal changes occurring within the proliferative lesions of a neoplastic system resulting in a series of qualitatively different lesions; lesions that *may* progress from benign to malignant to increasing malignancy. Viewed as a process, tumour progression is not obligatory. Indeed, the net directionality of lesions early in a tumour progression system is toward regression.

*Dysplasia* is a focal proliferation of cells with temporally restricted growth that shows an abnormal organisation of tissue (aberrant differentation) and randomly scattered cells with some of the features (cytologic atypia) of cells seen in primary cancer. Both features should be observed before the term dysplasia is used.

### The classes of neoplastic lesions

The sequential lesions of neoplasia may be readily divided into classes on the basis of growth characteristics, temporally restricted (not autonomous) or temporally unrestricted (autonomous or semi-autonomous), and whether lesional growth if confined to a single tissue compartment or involves two or more tissue compartments.*

### *Class I. The precursor lesions*

The growth of these lesions is temporally restricted and confined to the tissue compartment of origin. Initially, the lesions show preferential growth over the surrounding tissue, but growth ceases.

*Class IA. The initial lesion* The lesions are composed of an organised collection of neoplastic parenchymal cells associated with a histologically altered mesenchyme. The squamous papilloma, induced by a carcinogen in the mouse skin, is a prototype of an initial lesion. The lesions are biologically benign and are clonal in systems that have been studied (Burns, 1989). Of paramount interest, is the tendency for initial lesions to disappear by a programmed pathway of differentiation. If cancer is to develop in a neoplastic system, an indication of this possibility is the failure of an initial lesion to disappear. Initial lesions are numerous in most neoplastic systems and progression to the next lesional class, Class II, is quite rare. In addition to squamous papillomas, common acquired melanocytic nevi (ordinary moles), adenomatous colonic polyps (tubular adenomas), and hepatocyte nodules are examples of initial lesions. It may well be that the lesions designated and described in this paper as initial lesions are physiological and protective. Such lesions have been designated by Emmanuel Farber as 'clonal adaptation'. This concept is covered in the summary and discussion of this paper.

*Class IB. Aberrant differentiation in the initial lesion* Any lesion that fails to follow the programmed pathway of development, evolution, and disappearance via differentiation demonstrates aberrant differentiation, by definition. Such failure is usually accompanied by disorganisation of growth and cytologic abnormalities in the initial lesions or, less commonly, is only manifested by persistence of the initial lesion, rather than its disappearance. The form and behaviour of the initial lesion is the frame of reference for recognition and definition of the subsequent different types of Class I lesions. It should be emphasised that all lesions of a tumour progression series emerge focally in a preceding lesion. At the outset, the new lesion does not globally replace its predecessor. When tumour progression has reached Class IB, the lesional mesenchyme is noticeably different from the normal in routine histologic preparations. Such lesions may be stated to have both a neoplastic parenchyma and a neoplastic mesenchyme. Regression of Class IB lesions is seen and is similar to that seen in initial lesions, but most Class IB lesions are indolent. Progression to Class IC is uncommon. Prototypic lesions include: (1) Melanocytic nevi with focal abnormalities in the architecture of intraepidermal melanocytes (such as bridging of melanocytes from one rete to the next) when compared with the pattern of intraepidermal melanocytes in the initial lesion. (2) Persistent hepatocyte nodules with spontaneous proliferation of a subset of cells. (3) Squamous papillomas that do not regress and show mitotic activity above the basal layer. (4) Small persistent tubular adenomas of the colon.

*Class IC. Lesions showing dysplasia: aberrant differentiation and the appearance of some cells with some of the properties of cells seen in fully evolved primary cancer* The lesions show the aberrant differentiation of Class IB. Within the areas of aberrant differentiation atypical cells appear. Such cells are large with prominent and usually hyperchromatic nuclei. The individual cells may be indistinguishable from some cells seen in overt cancer; the lesions of Class III. However, there are differences. The atypical cells of Class IC tend to be separate from one another; they do not appear in contiguous array. After limited and focal growth in an initial lesion as a manifestation of tumour progression, growth again ceases. The lesions become indolent. Regression is not as common as in the initial lesion. Progression to Class II lesions is rare, but occurs. Representative and characteristic lesions include: (1) A melancytic nevus with dysplasia (the dysplastic nevus). (2) Persistent hepatocyte nodules with alteration of the cell cycle (3) Squamous papillomas with atypia (actinic keratoses in man). (4) Colonic adenomas with cytologic atypia.

### *Class II. The intermediate lesions*

The growth of the intermediate lesions is not temporally restricted, but the lesions are confined to the tissue compartment of origin. They may, however, focally extend into the immediately subjacent mesenchyme without evidence of the ability to grow in that mesenchyme. Class II lesions do not metastasise. As in previous steps in tumour progression, intermediate lesions arise as a focal event within a preceding lesional step, step IC, for example. Growth of intermediate lesions is usually quite slow but rarely ceases. The cells of Class II lesions are in contiguous array and there is a high probability of progression to the next lesional class. Prior to this step, the lesions of tumour progression tend to regress or become stable. The high probability of progression of Class II lesions to Class III is their most important biological property.

Lesional prototypes are readily characteristed by behaviour and histologic criteria. The prototypic lesions are confined to the tissue compartment of origin, but because of the appear-

*It is unlikely that any lesion of neoplasia is ever truly autonomous. Consequently, the terminology of temporally restricted . . . growth ceases after a time . . ., or temporally unrestricted . . . growth does not cease . . . is preferred. A tissue compartment is composed of an organised collection of one or more cellular phenotypes separated from other tissues by a basement membrane zone. Most tissue compartments are composed of a single cellular phenotype, as in the epithelium of the urinary bladder or the uterine exocervix. The human epidermis is an exception. During intrauterine life, three kinds of cells migrate into the keratinocytic epidermis: melanocytes, Langerhan's cells, and Merkel cells.

ance of the cells the lesions are commonly called by terms such as carcinoma *in situ*. This introduces major problems of nomenclature because Class II lesions do not have many of the properties of cancer. In fact, they lack its cardinal properties, except for temporally unrestricted growth. Consequently, I am hesitant to use terms such as carcinoma *in situ*, for its seems to me to be a contradiction in terms; the perfect oxymoron. Having said this, I have explained the quotation marks around the following lesions which exemplify Class II steps in neoplasia. (1) 'Squamous cell carcinoma *in situ*'. (2) 'Melanoma *in situ*'. (3) 'Carcinoma *in situ*' within an adenomatous colonic polyp. (4) Proliferative nodules within hepatocyte nodules (nodules within nodules). In spite of major semantic problems, some of these terms are so widely used, they are unlikely to be extricated from the lexicon of neoplastic terms and '*in situ* malignancy' will likely be a common term for Class II lesions.

### *Class III. Primary invasive cancer*

The lesions are temporally unrestricted in growth, grow in two or more tissue compartments, including the mesenchyme of the primary site, and may have competence for metastasis.

*Class IIIA: Primary cancer without demonstrable competence for metastasis* The excision of virtually any primary Stage I cancer results in cure of some percentage of cases. This percentage is frequently greater than 50% and may be higher. It seems unlikely that this significant percentage is due to extirpation at some propitious time; some limited time prior to the development of micrometastatic disease. An alternative possibility is that many primary cancers, even those showing growth in the mesenchyme of the primary site, may lack competence for metastasis and, possibly, would not have acquired such competence had they been left in place. Squamous cell carcinomas arising in actinic keratoses are rarely, if ever, associated with metastases. Carcinoma of the colon that does not extend to the muscularis propria is not associated with metastasis.

*Class IIIB: Primary cancer with manifest competence for metastasis* This class of lesions completely satisfies the definition of a primary invasive, tumourigenic cancer as the lesions have the ability to grow in three or more tissue compartments: the site of origin of the primary cancer, the mesenchyme of the primary site, and one or more distant sites. Squamous cell carcinoma of the skin that deeply invades the reticular dermis may metastasise. Carcinoma of the colon that extends through the muscularis propria is also associated with some incidence of metastatic disease.

### *Class IV. Metastases*

It is beyond the scope of this paper to discuss either the patterns of blood vascular metastasis or the biology of metastatic deposits. Two aspects of metastasis should be mentioned. First, a malignant neoplasm, whether primary or secondary, has a compelling growth preference over the surrounding tissues. If cells manifesting such a growth preference remained confined to a single site, the expanding tumour probably would become completely necrotic or would differentiate and cease growth. The continuance of the neoplastic sysem would seem to be dependent on the escape of cells from a site, either primary or secondary. Cells in a distant metastatic site, by definition, have full metastatic competence. Consequently, it seems quite reasonable to suggest that metastasis from metastasis is a routine aspect of neoplastic biology. It would then follow that therapeutic mechanisms that interrupt the metastatic pathway might have their most important effect on blocking metastasis from metastasis. The cells of a metastasis that could not escape from the site would surely die or differentiate. Second, a cardinal property of malignant neoplasia is the ability to grow in the mesenchyme of the primary and the secondary site usually without an intervening intact basement membrane zone. Malignant cells do not grow in the parenchyma of a secondary site; growth is in the mesenchyme. The mesenchyme of the distant site is foreign and may require a long period of tumour adaptation to the extracellular matrix (mesenchyme) of that site prior to significant growth.

The existence of different patterns and pathways of metastasis suggests that there may be subsets of cells in a primary cancer with properties that allow for their selection by specific organs and tissues. The following pathways are common, distinctive metastatic pathways.

*Lymphatic metastatic pathway without the blood vascular metastatic pathway* In all solid tumour systems (certainly the epithelial ones) some primary tumours are associated with metastasis to regional lymph nodes and, following therapy, are cured. Such behaviour again prompts questions of importance in tumour biology. Are there primary tumours that have produced a subset of cells capable of completing a lymphatic metastatic pathway, but incapable of completing a blood vascular pathway? If this should be the case, can such lymph node deposits acquire the capacity for blood vascular metastasis by further tumour progression in the lymph nodes? The lymphatic metastatic pathway could well be a 'route restricted metastatic pathway' (see 'organ restricted pathway', below). It is mentioned here to emphasise that some lymphatic metastases may not reflect progression of a primary tumour to complete metastatic competence.

*Lymphatic metastatic pathway with concurrent or subsequent blood vascular metastases* The concurrent presence of lymphatic and blood vascular metastases indicates, in all likelihood, that a tumour has a subset of cells capable of completion of both kinds of metastatic pathways. It may well be that cells capable of completing the blood vascular pathway can also complete the less complex lymph vascular pathway. When blood vascular metastases follow, by some time (more than a year, for example), lymph node metastases, tumour progression may have occurred within the lymph node deposit. An alternative possibility posits that blood vascular metastases occurred at the time of the lymph vascular metastases, but their manifestation was delayed. Such a delay could be related to adaptation of tumour cells to a foreign (distant) extracellular matrix.

*Blood vascular metastases without demonstrable lymph vascular metasatases* Such metastases may reflect a paucity, relative or absolute, of lymphatic vessels at the site of the primary tumour. Primary tumours with an exuberance of blood vascular angiogenesis may have the majority of cells capable of metastasis entering venules in preference to lymphatics.

*Organ restricted metastatic pathways* Metastases may, from time to time, be limited to a single organ or tissue. Common restricted pathways include cutaneous metastases and metastases to the lungs or brain. Such patterns of spread may reflect the existence of cells only capable of growth in the mesenchyme specific to a given organ or tissue. Such specific pathways have been clearly demonstrated experimentally (Morikawa *et al.*, 1988; Zetter, 1990).

### Melanocytic neoplasia as a model for tumour progression

### *Class I. The precursor lesions. The melanocytic nevus. Growth is temporally restricted.*

*Class IA. The initial lesion. The common acquired melanocytic nevus.* Growth, differentiation and disappearance are evident in the clinical behaviour of the ordinary mole and in the histologic changes that correlate with its distinctive life history. Melanocytic nevi appear between the 6th month and 2nd year of life. Initially, the lesion is a flat, tan dot. Enlargement occurs slowly at its periphery and, when the lesion is 2–3 mm in width, it gradually becomes elevated. With further elevation pigment synthesis diminishes, and the mole becomes a flesh coloured tag of skin. Following this the lesion may slowly flatten and disappear. The whole process covers one to four decades. We have observed individual moles progress from flat, tan-brown to soft skin tags in 4–6 years, but the appearance and disappearance of the entire population of moles of a given individual covers several decades. Normal people have relatively few moles by the 6th decade of life. Interestingly, patients who have had melanoma may show persistence of their melanocytic nevi into the 6th or 7th decade. Histologically, the appearance, growth and disappearance of nevi is as distinctive as the clinical story. An increased number of melanocytes in the basal epidermal layer is noted in the earliest visible lesions. The subjacent mesenchyme is altered, appearing as a dense eosinophilic band. During the period of peripheral enlargement of the nevus, melanocytes form nests at the tips of rete ridges; an early manifestation of differentiation. With nest formation the parenchymal neoplastic cells seem to extend into the dermis. As the cells appear in the dermis they are surrounded, individually or in small clusters, by a basement membrane zone (Schmoeckel *et al.*, 1989; Lea & Pawlowski, 1986; Yaar *et al.*, 1988); traverse of the basement membrane zone is not accomplished by lysis of that zone, as in 'malignant' traverse of a basement membrane zone (Liotta *et al.*, 1987). In the dermis, the outermost cells continue to synthesise pigment and are tyrosinase positive. Deeper, the cells round up and become tyrosinase negative. Cells then elongate and differentiate along Schwannian lines (Aso *et al.*, 1986). The elongate Schwannian cell, not the epidermal melancyte, may be the terminally differentiated cell of neural crest origin. Finally, a delicate neuromesenchyme is formed and the lesion disappears. This life history of a nevus and its correlated histology is a prototype of the expected behaviour of an initial lesion. Deviation from the expected path is the abnormalcy. Some lesions do not follow the complete differentiation pathway and become stable; arresting at different stage of differentiation. The failure to differentiate and disappear is one manifestation of progression to step IB.

The appearance and behaviour of normal nevi and all Class I lesions have been depicted in a series of colour photographs (Greene *et al.*, 1985). The histology of the various precursor lesions has also been described in detail (Clark *et al.*, 1984; 1990).

*Class IB. A melanocytic nevus with aberrant differentiation* Persistence (in and of itself) of a common acquired melanocytic nevus without any subsequent differentiation and without a manifest pattern of abnormal melanocytic growth may be regarded as an aberrant phenomenon; a subtle expression of aberrant differentiation. Aberrant differentiation is usually manifested by an area of melanocytic growth at the periphery of a common mole. The margin of a mole with such growth shows an irregular flat area with an indistinct border. The area may involve all or a portion of the border of the mole and its presence adds asymmetry to the initial lesion and frequently variation in colour (Greene *et al.*, 1985). Histologically, the area shows an increase in the number of melanocytes with some elongation of hyperpigmented rete (Clark *et al.*, 1984). Such peripheral growth stimulates the beginnings of the initial lesion. However, in areas of such growth the melanocytes do not form orderly nests at the tips of rete, but, instead, elongate clusters that tend to bridge from one rete to the next. Most of the rete are surrounded by a compact band of eosinophilic connective tissue; abnormality of the extracellular matrix is evident early in the flawed differentiation of an initial lesion.

*Class IC. A melanocytic nevus with dysplasia* Clinically IB and IC lesions are similar. The areas of dysplasia are usually limited to a portion of the nevus and are characteristic histologically. The dyplastic nevus is distinguished by the superimposition of atypical melanocytes upon the nevus with aberrant differentiation. The atypical cells are usually admixed with the aberrant melanocytes lacking atypia at the periphery of a normal nevus. The atypical cells may be $2\times-4\times$ the size of normal melanocytes, have large nuclei, and are numerous and readily seen (Rhodes *et al.*, 1988). An occasional atypical melanocyte in an area of aberrant differentiation does not warrant a diagnosis of dysplasia. The atypical cells do not grow contiguously with each other, as a rule, but one may see two or three atypical melanocytes in the basilar region of the epidermis adjacent to each other; contiguous growth of atypical cells is the hallmark of Class II lesions. In some instances the atypical cells are large, epithelioid melancoytes and these cells have an abundance of tan cytoplasm and, usually, large nuclei. Such cells may be at the edge of nevi, above dermal papillae, at the edges of rete, or at the tips of rete. Their distribution is random. Some atypical melanocytes may be seen in the subjacent dermis. The dermis in areas of dysplasia is distinctive. Narrow bands of brightly eosinophilic, acellular collagen commonly rim the dermal-epidermal interface covering a distance of several rete and papillae; an extracellular matrix change that has been termed concentric eosinophilic fibroplasia. Less commonly seen, but even more characteristic, is another connective tissue change called lamellar fibroplasia. Lamellar fibroplasia frequently appears at the tip of a rete ridge. Such rete have ill defined nests of melanocytes at their tips. These melanocytes are not arrayed as those in a normal nevus. They may be spindled and a cluster of them is ill-defined. The cells blend with the subjacent lamellar fibroplasia and contribute to its formation. The lamellar fibroplasia is triangular in outline in a two dimensional plane, but doubtless a cone in three dimensions. The lamellar fibroplasia is composed of delicate strands (cross section of a plate if it were seen in three dimensions) of collagen alternating with elongated nuclei. Small granules of melanin may be seen on either side of the nuclei. One gets the impression that the cells of lamellar fibroplasia are derived from epidermal melanocytes that synthesise both collagen and malanin and are differentiating, to some extent along Schwannian lines, for the stacked, plate-like structure resembles a Wagner-Meissner corpuscle. Lamellar fibroplasia also strongly suggests parenchymal tumour cell-extracellular matrix interaction. In addition to the connective tissue changes there are always small collections of lymphocytes about the blood vessels of the superficial vascular plexus (Clark *et al.*, 1990).

The atypical cells have many antigenic characteristics of 'melanoma cells' seen in Class II and Class III lesions of tumour progression (Aronson *et al.*, 1988). Some studies of the antigenic profile of tumour progression stages in melanocytic neoplasia show distinctive differences (Holzmann *et al.*, 1987; Elder *et al.*, 1989). Abnormalities of cellular DNA content and random chromosomal abnormalities have been reported in dysplastic nevi (Bergman *et al.*, 1988; Newton *et al.*, 1988; Parmiter *et al.*, 1988). The lesions are usually stable, but have some risk of progression to the next class of lesions.

*Class II. The intermediate lesions*† *'in* situ *melanoma' and radial growth phase melanoma: growth is not temporally restricted but is confined to the tissue compartment of origin or barely extends into the subjacent dermis. Actual growth in the mesenchyme apparently does not occur*

*Class IIA. Malignant melanoma* in situ The prototype of this class is the kind of lesion that may evolve into an overt malignant melanoma of the superficial spreading type (a Class III lesion). Remnants of preceding Class I lesions may or may not be observed histologically in Class II lesions. The cells of Class II lesions are large, epithelioid and have an abundance of cytoplasm filled with tan, finely divided melanin. The cells tend to be contiguous with each other when

†The terminology problem is most difficult with intermediate lesions for they lack many of the cardinal properties of biological cancer. The terms melanoma *in situ* and radial growth phase melanoma are well established in the literature. Their use does not imply cancer, as herein defined. The lesions behave and are intermediate between the relatively stable precursor lesions and fully evolved cancer.

compared with the atypical cells of a dysplastic nevus. The nuclei of the distinctive cells of Class II lesions are large and hyperchromatic. Nucleoli may be prominent. Although the individual melanocyte is atypical, having the just described features, the overall appearance of the melanocytes is one of homogeneity; variation from cell to cell is not prominent in most cases. Cells are disposed in nests and as individual cells at all levels of the epidermis, including the stratum granulosum. Individually disposed cells may dominate over those in nests. The pattern of the cells may simulate Paget's disease of the breast.

*Class IIB. Primary malignant melanoma in the radical growth phase* This class of neoplastic lesions shows the histology of *in situ* melanoma and focal extension of similar cells into the subjacent papillary dermis. The cells in the dermis are disposed as individual cells and as small clusters. No focus of dermal cells seems to have a growth preference over the others. Growth apparently does not occur in the dermis, but the cells in that location arrive there from the epidermis, which is apparently the site of tumour cell division in this stage in tumour progression. The dermal cells are usually surrounded by an intact basement membrane zone, which seems to be continually formed around the individual cells and the small nests of cells. Of course, such histologic pictures are usually interpreted as invasion, but in spite of the presence of 'melanoma cells' in the dermis metastases do not occur. We have followed 149 such cases for $>8$ years without any evidence of metastasis[‡]. This observation brings into focus a critical point in tumour biology: invasion as determined by the routine methods of pathology does not necessarily mark a tumour as having metastatic competence. Invasion and metastasis are, to some extent, disparate properties. Invasion, as exemplified by radial growth phase melanoma, precedes and is necessary for metastatic competence. The next tumour progression step, actual growth in the mesenchyme, is a marker, albeit an imperfect marker, of metastatic competence. The cells that extend into the dermis in the radial growth phase initiate a form of tumour-matrix interaction that results, in due course, in competence for growth in that matrix (dermis), the expression of which is vertical growth phase.

*Class III. Primary malignant melanoma with manifestation of growth in the mesenchyme of the primary site*

Progression to overt cancer is not the end of tumour progression. Primary melanomas illustrate continuation of tumour progression within an established cancer better than most neoplastic systems. One can frequently observe, clinically and microscopically, evidence of tumour progression in primary melanomas. This is due, in part, to changes in pigment synthesis with the sequential appearance of different populations of cells in the primary neoplasm. For example, a pink nodule may appear within a previously brown-black melanoma. Thus, tumour progression is manifested, in most melanomas, by a distinctive biphasic growth pattern. In the first phase the tumours slowly enlarge along the radii of an imperfect circle; net growth is at the periphery of the lesion. This pattern of growth is the radial growth phase of melanoma and has just been discussed as the second part of the Class II lesions. The second phase of growth, the vertical growth phase, of the primary lesion is characterised by focal, dermal growth somewhere within the initial growth phase. In due course the second growth phase forms an expansile nodule; a pattern of growth that is similar to that seen in a metastasis and one that may be a portent of metastasis. The two growth phases of a primary melanoma are not only different in their macroscopic (clinical) and microscopic presentation, but have distinctive antigenic differences. In addition, cells derived from the different growth phases have different *in vitro* characteristics. Elder *et al.* have shown that radial growth phase melanomas have an antigenic profile similar to dysplastic nevi, while vertical growth phase melanomas present an antigen profile similar to metastatic melanoma (Elder *et al.*, 1989). Herlyn *et al.* have shown that cells derived from radial growth phase melanomas rarely produce permanent cell lines and those few cell lines that have been maintained are not tumourigenic in nude mice (Herlyn *et al.*, 1987).

[‡]All but two of these cases have been followed for $>10$ years. One patient died of myocardial infarction after 8 years and three were lost to follow-up after 8 years.

*Class IIIA. Primary malignant melanoma in the vertical growth phase without manifest competence for metastasis* Some 63% (207/328) of primary, vertical growth phase melanomas do not show metastatic disease after 8 years of follow-up. There are many possible explanations for this lack of manifest competence for metastasis. The biological inferences of attributes that are independently predictive of survival will be discussed in the following section (vertical growth phase melanomas with metastases). Among the diverse explanations for the lack of metastasis there could well be a subset of tumours capable of growth in the mesenchyme of the primary site, but incapable of completion of any step in a metastatic pathway, except for invasion and some motility in the dermis. If such a subset can be identified and characterised, it would clearly demonstrate that invasion and the other properties required for metastasis are not acquired as a group of attributes, but as properties acquired seriatim. At present, one can record those attributes likely to be associated with long periods of disease free survival. The following is a list of such attributes derived from the data base of the Pigmented Lesion Group of the University of Pennsylvania. It should be here emphasised that the list is for primary melanomas in the vertical growth phase. Radial growth phase melanomas have not been associated with any form of metastasis in our experience and are eliminated from a discussion of metastatic competence.

Vertical growth phase melanomas having all of the following characteristics have not been associated with metastases in our data base. Thirty-two cases have had all of the attributes and none have shown metastases.

(1) Mitotic rate: $0\,mm^{-2}$.
(2) The presence of brisk tumour infiltrating lymphocytes.
(3) Thickness: $<1.70\,mm$.

*Class IIIB. Primary malignant melanoma in the vertical growth phase with metastasis* The following attributes are independently predictive of survival in Stage I primary melanomas and, consequently, are the best candidates for a positive or negative relationship to metastatic pathways. (1) *Mitotic rate* $mm^{-2}$ A quite attenuated list of steps in a metastatic pathway would include: (a) the number of cells produced by a primary neoplasm in a given time; (b) the number of cells migrating away from the primary site; (c) the number of cells entering a venule or a terminal lymphatic; (d) the number of cells surviving in the blood or lymph vascular pathway; (e) the number of cells escaping from vascular pathway; and (f) the number of cells surviving and replicating at the metastatic site. Regardless of how long one makes such a list of steps in the metastatic pathway, it must ultimately be related, in some way, to the rate of production of the cells of a primary melanoma. Attesting to this statement is that no other attribute has the survival predictive power of mitotic rate (Clark *et al.*, 1989). In vertical growth phase melanomas, 95% (39/41) of patients with a mitotic count of $0\,mm^{-2}$ survive $>8$ years, while only 38% (26/68) with a mitotic count $>6\,mm^{-2}$ survive $>8$ years (Figure 1). At 10 years follow-up, 206 patients who have been disease free had a median mitotic rate of $1.1\,mm^{-2}$, while patients dying of disease during the 10 year period had a median mitotic rate of $6.2\,mm^{-2}$ (Data Base of the Pigmented Lesion Study Group, 1991). (2) *Tumour infiltrating lymphocytes* The presence of a brisk infiltrate of tumour infiltrating lymphocytes (TIL's) is associated with death due to metastatic disease in

**Figure 1** Stage I vertical growth phase melanoma. Mitotic rate (no./sq.mm) – outcome. Dead of disease or alive at 8 years. ■, DOD; □, Alive.

**Figure 3** Stage I vertical growth phase melanoma. Thickness – outcome. ■, DOD; □, alive.

only 11% (6/52) of cases, while the absence of tumour infiltrating lymphocytes is associated with a 41% (44/108) mortality due to disease (Figure 2). To qualify as tumour infiltrating lymphocytes the cells must actually infiltrate and disrupt the tumour. Lymphocytes at the base of the tumour, regardless of density, do not influence outcome. One tends to immediately attribute the significance of TIL's to a cytotoxic immune function. However, there are other possible, even plausible, roles for the effect of tumour infiltrating lymphocytes. A brisk infiltrate of lymphocytes could inhibit cell motility or block entry into vascular spaces. (3) *Tumour thickness* Increasing tumour thickness is clearly related to increasing mortality (Figure 3). The significance of increasing thickness is independent of other attributes, and, specifically, independent of mitotic rate. Why is thickness significant? It could be a surrogate for time during which futher tumour progression occurred. New cell populations with enhanced

**Figure 2** Stage I vertical growth phase melanoma. Tumour infiltrating lymphocytes. Outcome dead of disease or alive at 8 years. ■, DOD; □, alive.

capacity for metastasis could have appeared during the time required for increasing thickness. Thick vertical growth phase tumours have chromosomal abnormalities, usually at chromosomes #1, #6 and #7 and genetic alterations appear in parallel with increasing biological aggressiveness (Nowell, 1989; Parmiter & Nowell, 1988). Increasing thickness also results in an increasing interface with the vascular bed. If one treats the vertical growth phase as a sphere of tumour cells, doubling of the thickness of the tumour increases its outer surface, the surface of the sphere, by 4-fold. If one considered the interstices of the tumour, the interface with the vascular bed is increased many fold. A corollary of increasing interface with the vascular bed is an increasing interface with the extracellular matrix. We have observed that increasing thickness and invasion of the reticular dermis (Level IV invasion) is associated with a decrease in formation of the basement membrane around the tumour cells (unpublished observations). Tumour cell interaction with the nonbasement membrane extracellular matrix is then possible. Such interaction may be necessary for adaptation to and continuing growth in the extracellular matrix and may also be related to acquisition and manifestation of metastatic competence. In summary, increasing thickness is correlated with progressive chromsomal abnormalities; an increasing interface with the vascular bed (probably associated with angiogenesis); and progressive interaction with the extracellular matrix. The expression of many tumour cell properties could be the result of signals between the abnormal tumour cells and the extracellular matrix, a reciprocal interaction. (4 & 5) *Anatomic site of the primary and sex of the patient* Female patients do better than male patients and patients with extremity lesions (excluding volar-subungal lesions) do better than those with axial lesions (head, neck, and trunk). Divergent behaviours that is site dependent again brings up the possibility of influence by matrix-tumour interaction. The dermis varies in form at different sites, and may well vary between the two sexes. Such variations could influence the kind of tumour evolving at a given site and in a given sex; the phenomenon of topooncogenesis (to alter Edelman's term of topobiology and apply it to the abnormal life form that is cancer) (Edelman, 1989). (6) *Histologic foci of complete regression* Such foci could be due to immunoselection of a population of cells that has escaped the immune system. Prehn has postulated another mechanism for the adverse significance of regression (Prehn, 1990). He proposes that during the evolution of a melanoma two properties concerning lymphoid infiltrates progress independently. Tumour cells give off an attractant for lymphocytes and this substance may decrease with progression. Secondly, tumour cells may be dependent, initially, on lymphoid cell products for growth and this dependence decreases with progression. Prehn further suggests that some melanoma cells may lose the power to attract lymphoid cells before they lose growth dependency. Regression may then occur, but other parts of the clone of cells in the vertical growth phase may lose growth dependency along with their ability to attract lymphoid cells. In such an instance, regression may mark a poor prognosis because cells adjacent to the regression can no longer attract lymphocytes and no longer need to do so in order to grow; they have escaped dependence on a part of their microenvironment.

From the foregoing considerations one may derive a list of attributes associated with metastatic disease. Fifteen patients in our data base have had all of the following attributes and all have developed metastatic disease.

(1) A mitotic rate $>6\,mm^{-2}$.
(2) Absence of tumour infiltrating lymphocytes.
(3) Thickness $>3.60$ mm.

### *Metastasis*

*The different metastatic pathways* In cutaneous melanocytic neoplasia there are three important observations suggesting the existence of biologically different metastatic pathways. First, of those melanomas that metastasise, 18% (see following discussion) involve regional lymph nodes without subsequent dissemination of any kind. In contrast, metastasis of melanoma to any other site, with few exceptions, is followed by death due to disseminated disease. Second, the terminal lymphatic of the skin usually does not have a basement membrane zone. The lymphatic endothelium abuts directly against the dermal mesenchyme. Entry of mobile tumour cells into the terminal lymphatic could be a much simpler task than entry into a venule. Third, there exists an uncommon form of melanoma that seems to be a natural experiment with regard to a distinctive metastatic pathway. Reed has described a Spitz type of minimal deviation melanoma with metastasis to regional lymph nodes without dissemination (Reed, 1988). Smith *et al.* have reported six cases of metastasis of 'malignant Spitz nevi' to regional lymph nodes without subsequent disease. I have seen four similar cases. The primary lesions are large, thick tumours that frequently involve the subcutis. The cells in the lymph nodes are virtually identical with those in the primary. These rapidly growing tumours of young people (essentially, the first two decades of life) seem to have the capacity for lymphatic metastasis but do not seem to be able to successfully compete any other metastatic pathway. this may be related to the lymphatic invasion frequently seen in benign Spitz tumours (Howat & Variend, 1985). The relative incidence of the different metastatic pathways in our data base is shown in the following list. The data are based upon 328 vertical growth phase melanomas. In this data set there were 121 (37%) cases with some form of metastasis during a period of $\leqslant 8$ years. During that follow-up period, the remaining cases (207) did not show metastases.

*Lymphatic metastatic pathway without the blood vascular metastatic pathway* 22/121 (18%).

*Lymphatic metastatic pathway with concurrent or subsequent blood vascular metastases* 47/121 (39%).

*Blood vascular metastases without demonstrable lymph vascular metastases* 52/121 (43%).

*Organ-restricted metastatic pathways* Occasionally ($<1\%$) metastases seem to be limited to a single organ. In melanoma this is most commonly seen as cutaneous metastases. Occasionally, even when beyond the region of the primary, removal of cutaneous metastases, as they appear, is followed by cessation of the phenomenon and no further disease is manifest. One gets the impression that the tumour cells do not have the capacity for growth in any mesenchyme other than the dermis, the original mesenchyme of the primary melanoma.

### Tumour progression in other neoplastic systems

It is not an objective of this discussion of the analogies between the lesions of tumour progression in diverse neoplastic systems to precisely describe all of the sequential lesions of each system. We would like to show that neoplastic systems are remarkably similar, and these very similarities form a conceptual framework for neoplasia and may indicate research directions of importance in the understanding of some of the basic problems in neoplasia. For example, the initial lesion of neoplasia is a focal benign growth that is probably clonal (Burns, 1989); is largely independent of the inductive mechanisms (see following discussion); and is a lesion that usually disappears by a programmed pathway of differentiation. The molecular biology of this primary response to a carcinogen – growth, cessation of growth, and disappearance – has not been extensively investigated. The failure of a given lesion to follow the programmed pathways of an initial lesion (Class IA) – growth, cessation of growth, and disappearance – is the first step in a pathway to cancer; the first step in carcinogenesis. Knowledge of the principles of tumour progression is at the foundation of the present control of mortality due to cancer. Such control is largely based upon knowledge of cancer risk afforded by the existence of some of the Class I lesions. Those individuals identified as having a high risk for any given form of cancer must then have appropriate education, follow-up and therapy. In melanoma, for example, Masri *et al.* have shown that melanomas diagnosed in follow-up of patients with a family history of melanoma had an average thickness of 0.52 mm, while the melanomas of the index cases of that study had a average thickness of 1.44 mm (Masri, 1990). We would also like to show that study of the individual lesions appearing relatively early in tumour progression permits biological inference about cancer development that cannot be derived from the study of cells emerging at the end of tumour progression (cells derived from fully developed primary cancers and from metastases).

### *Class I lesions. The precursor lesions*

*Cutaneous keratinocytic neoplasia* Demonstration of reproducible behaviour in the seemingly diverse lesions of neoplasia requires an experimental system (Klein-Szanto, 1989). Virtually all human neoplastic systems evolved while the inductive mechanisms are continually active. This is obviously the case when the inductive mechanisms involve light and cutaneous neoplasia. Light impacts upon the skin throughout most of the life span of man. Consequently, observations of coordinated behaviour of cohorts of lesions are confounded by the emergence of sequential crops of lesions at all stages of evolution and regression. If one observes, on a single occasion, a 40 year old red-haired, freckled patient, who has had significant sun exposure, the cutaneous neoplastic lesions of the keratinocytic system will be quite diverse. Benign keratoses, actinic keratoses, and, perhaps, a squamous cell carcinoma will be present. Sorting out the histogenetic and sequential relationships between the many lesions based upon a single observation is likely to be impossible. The problem is similar to the full appreciation of a complex musical canon of eight parts when one hears only the middle of the composition. An animal experiment inducing a neoplastic system permits one to play one part of the canon of neoplasia from the beginning to the end. Experiments with syngeneic animals with controlled dosages of carcinogens produce lesions that behave in a uniform fashion. From such studies one may reasonably infer that most human neoplastic systems have lesions that are analogous with the reproducible lesions produced in experiments. A further complication of the understanding of tumour progression, especially in man, is the continuing action of the inductive process on extant lesions. For example, nothing is known about the effect of UV irradiation on Class II lesions. Does it inhibit growth, enhance growth, induce a new clonal tumour progression step, or is it without effect?

The initial lesion in mouse skin induced by a chemical carcinogen and croton oil is a squamous papilloma. Under defined conditions such lesions grow, become indolent and disappear via a pathway thought to be due to differentiation. A similar papillary lesion due to HPV 16 or 18 (and some other subtypes) occurs on the human genital skin. Many of these papillary lesions, termed bowenoid papulosis, also spontaneously disappear. The initial lesions, in animals, due to UV-light are also squamous papillomas. It is quite remarkable that such different inductive mechanisms evoke the same initial response: a papillary hyperplasia that tends to regress and disappear. The common initial lesion of keratinocytic neoplasia in human skin is a benign keratosis, a circumscribed area of thickening of the epidermis which is not papillary in form. The lesions of aberrant differentiation (Class IB) and dysplasia (Class IC) are characterised by persistence of the intial lesion, mitotic activity above the basal layer and, in Class IC, atypical keratinocytic hyperplasia. An actinic keratosis is the prototype of a Class IC lesion and is the prototype of a lesion that may progress to squamous cell carcinoma. It must be emphasised that most actinic keratoses, like other Class IC lesions are indolent, end stage lesions without a future.

*Hepatic neoplasia. Microscopic islands and hepatocyte nodules* Hepatocyte nodules arise in microscopically visible islands of altered hepatocytes. The cells of the islands show reproducible histochemical and biochemical changes (Farma & Sarma, 1987). Some are of the opinion that microscopic islands may lead to cancer without the prior appearance of hepatocyte nodules (Williams, 1980). The nodules are macroscopically visible, focal proliferations of hepatocytes that are different from and compress the surrounding liver. The phenotypic differences include arrangement and architecture of hepatocytes, blood supply, cytologic and histologic appearance and biochemical properties. Metabolic patterns are remarkably uniform from one model of hepatocarcinogenesis to another and, largely, from one nodule to another, including persistent nodules. Under appropriate experimental conditions the hepatocyte nodules differentiate and disappear, leaving a liver that is essentially normal in appearance. Recently it has been shown that the reversion of hepatocyte nodules to normal liver is via a complex genetic program of differentiation. The programmed differentiation '... points to the physiological nature of many of the early steps in the carcinogenic process' (Farber & Sarma, 1987; Farber, 1990). One of the reasons for using analogy as an investigative tool is to recognise important events by their clear portrayal in one system. The demonstration and study of the disappearance of the initial lesion in neoplasia has been done consistently in the hepatic system and should be regarded as a prototype of behaviour of initial lesions. The initial lesions, in Farber's view, are not pathology but a physiological response, he has termed clonal adaptation (Farber, 1990). Search for the initial lesion in another system may reveal its presence more subtly manifested. The gradual differentiation and obscure disappearance of the common acquired melanocytic nevus may be recognised in the light of differentiation of the hepatocyte nodule. If a hepatocyte nodules is to progress, it fails to differentiate and shows a focal proliferation of a subset of cells. An additional step, one quite likely to lead to carcinoma, is the proliferation of a subset of cells showing an alteration of the cell cycle within persistent hepatocyte nodules. Failure of differentiation and nodules within nodules are analogies with Class IB and IC lesions in other neoplastic systems.

*Colonic neoplasia* Vogelstein and his colleagues have shown that three sequential adenomas, early, intermediate, and late, precede carcinoma of the colon. Such designations parallel polyp classifications by pathologists. Late adenomas usually show significant cytologic atypia. Adenomas regress as do melanocytic nevi, squamous papillomas and hepatocytes nodules (Feinberg *et al.*, 1988). As a rule the sequential series of qualitatively different polyps is accompanied by progressive increase in size of the lesions. The colonic polyp sequence is easily fitted into the Class IA, IB, and IC scheme of this presentation. The parallels between colorectal cancer development and other neoplastic systems are easily seen. Vogelstein's work has provided, in addition, evidence that progression from the polyp sequence to carcinoma to metastasis is associated with the sequential accumulation of genetic abnormalities (oncogene activation and inactivation of suppressor genes ) (Fearon *et al.*, 1990). His findings do not explain the behaviour of the sequential lesions of neoplasia, but should be a stimulus for the orderly investigation of neoplastic development in other systems. Investigation from the beginning, not from the end. If the lesions of neoplastic systems are analogous, the cellular mechanisms of their development should be similar.

*Class II lesions. The intermediate lesions*

*Cutaneous keratinocytic neoplasia* The prototype of intermediate lesions is squamous cell carcinoma *in situ* in a stratified squamous epithelium such as the epidermis or uterine cervix. Such lesions show progressive growth, which is usually quite slow. Histologically, they differ from Class IC lesions in that all of the cells of the lesion are atypical and are in contiguous array throughout the entire thickness of the epithelial surface. The characteristic lesions are entirely above the basement membrane zone and are not associated with any apparent ability to metastasise. Additional lesions will also show cells below the basement membrane zone. These cells are disposed individually, as a rule, and are also apparently unable to metastasis. Lesions with a few cells below the basement membrane zone do not have the capacity for growth in the mesenchyme; a capacity that may be one requirement for acquisition of metastatic competence.

*Hepatic neoplasia* The detailed study of the later steps of cancer development in the rat liver has not been done. The extensive investigations of hepatocarcinogenesis have concentrated on initiation, promotion and the earliest steps of tumour progression; steps preceding cancer. In fact many studies of experimental carcinogenesis seem to treat the appearance of invasive cancer as an end point. The complex events occurring within what we here term Class II and Class III lesions have not been the subject of detailed inquiry in experimental cancers. However, a group of lesions designated as nodules within nodules, while not overtly invasive, does progress to invasive cancer. Consequently, they seem to be analogous with Class II lesions. Transplantation of the lesions designated as nodules within nodules to the spleen of syngeneic animals is followed by progression of the transplanted lesion to overtly invasive cancer (Farber & Sarma, 1987).

*Colonic neoplasia* Late adenomas commonly show areas that in other neoplastic systems would be called carcinoma *in situ*. One may also see, in these late adenomas, areas of extension below the basement membrane zone. Actual growth apparently does not occur in the mesenchyme of the site. As long as such extensions below the basement membrane zone do not extend to the lamina propria, the lesions are not termed carcinoma for no metastases occur (Plaz, 1978). Such lesions are precise analogies with radial growth phase melanomas. In colonic neoplasia the term carcinoma is not used for lesions that have no potential for metastasis. Obviously such lesions are prototypes of the Class II lesions as discussed in this paper.

*Class III lesions. Primary invasive cancer with manifestation of growth in the mesenchyme of the primary site*

These lesions are the lesions with some potential for metastasis. As a rule the diverse metastatic pathways that seem manifest in melanocytic neoplasia are not discussed at length in other forms of neoplasia. Study of different neoplastic systems, however, shows clear analogies with melanocytic neoplasia.

*Cutaneous keratinocyte neoplasia* Most squamous cell carcinomas are superficial and arise in actinic keratoses. Such lesions do not metastasise, for practical purposes, and are similar to radial growth phase melanoma or early vertical growth phase melanoma. The carcinomas that deeply invade the reticular dermis, or carcinomas near mucocutaneous junctions will show metastasis, but not commonly. Progression to full metastatic competence is not as common in cutaneous squamous cell carcinoma as it is in melanoma.

*Colonic neoplasia* Those tumours that invade only into the submucosa, without involvement of the muscularis propria, are not associated with metastatic disease. Such lesions are similar to radial growth phase melanoma. Deeper involvement of the bowel wall and extension through the bowel wall parallel some aspects of the varying prognosis of vertical growth phase melanoma. Other prognostic attributes, such as mitotic rate and tumour lymphocytic infiltrating lymphocytes, are not routinely recorded in colon carcinoma. Even with lymph node involvement, however, some colon carcinoma patients survive. There always seems to be some subset of patients with primary colon carcinoma whose tumour has not acquired metastatic competence.

### A tabular comparison of tumour progression in different neoplastic systems

Class I and Class II lesions of the different neoplastic systems are compared in tabular form (Table I). In the same table the relative incidence of the different metastatic pathways of melanoma is shown.

### Neoplastic systems without demonstrable precursor (Class I) lesions: direct tumour progression

Individual primary cancers sometimes appear without manifest precursor lesions in both human and experimental cancer. Foulds has termed the phenomenon direct tumour progression. He stated, 'It seems to be a general rule, to which no clear exception has been demonstrated, that in every tissue in which a particular kind of malignant tumours *usually* develops along an indirect path through visible intermediate lesions, a similar kind of tumour *sometimes* emerges with its definitive characters established from the beginning and without tranversing any intermediate lesion during its clinically evident course' (Foulds, 1969). The phenomenon is rare in experimental neoplasia in syngeneic animals, but seems to be more common in man. In melanoma, for example, some 50–60% of primary melanomas have a dysplastic nevus contiguous with the primary melanoma or have dysplastic nevi present elsewhere on the skin. About 90% of patients bearing melanoma have some form of precursor lesion (Class I) either adjacent to the primary melanoma or somewhere on the skin. Regardless of the precise figures, there is some subset of primary melanomas that develop without evident precursor lesions. There are several possible explanations for this apparent manifestation of direct tumour progression. (1) *The growth of the primary melanoma ablates the evidence of the precursor* This doubtless occurs. Sagebiel has shown that small (thin) melanomas of the superficial spreading and nodular type are more commonly associated with adjacent precursors than are thicker tumours. We have made similar observations on melanomas in our data base. In melanomas of the superficial spreading and nodular types, the incidence of melanocytic precursor lesions adjacent to the primary melanoma by thickness groups is as follows. The first figures are those of the University of Pennsylvania Pigmented Lesion Group and the second those of the University of California at San Francisco (Data Base of the Pigmented Lesion Study Group, 1991; Sagebiel, 1991).

| | | | |
|---|---|---|---|
| (a) $<0.76$ mm | 58% | (121/207) | UCSF ... 64% (429/670) |
| (b) 0.76–1.69 mm | 47% | (65/138) | UCSF ... 64% (419/639) |
| (c) 1.70–3.60 mm | 42% | (40/95) | UCSF ... 44% (198/401) |
| (d) $>3.60$ mm | 26% | (14/53) | UCSF ... 34% (57/169) |

(2) *Direct tumour progression actually occurs* in the sense that something comparable to *in vitro* transformation of a putatively normal cell in a single cell generation occurs *in vivo*. A comment on this possibility with compelling evidence one way or the other approaches the impossible. Epidemiologic evidence is strongly against the possibility. One may surmise that if such a pristine example of direct tumour progression did occur, it would be seen in all age groups and perhaps more commonly in the young; the latter having cell systems quite susceptible to injury. As a matter of fact, melanomas occurring without any precursor anywhere on the skin or contiguous with the tumours are found in older patients. Such an observation suggests a long period of induction, not a 'hit' transforming a cell in one or a few generations. *In vitro* studies provide further evidence against an idealised form of tumour progression. Putatively normal cell lines usually require several sequential events, such as oncogene activation, for malignant transformation (Nicholson, 1987). (3) *Structurally atypical cells, similar in appearance to some cells in fully evolved primary cancers, develop without there being a clinically manifest lesion* This concept posits a concatenation of events over time in single cells leading to cytologic atypia, without the formation of recognisable Class I lesions; in one sense such a view implies something akin to tumour progression could occur in single cells. Such cells may be numerous, but they are separate from each other. The first line of evidence supporting the concept that scattered atypical cells not associated with a demonstrable lesion afford a melanoma risk is that atypical cells within a demonstrable precursor are the primary source of risk of these lesions. For example, in studying the risk of melanoma development when patients have precursor lesions, the greatest risk (400 × the baseline population risk) is afforded by those having a family history of melanoma *and* histologic dysplasia in two or more nevi (Green *et al*., 1985). The histologic dysplasia associated with such a risk was characterised by the presence of readily recognisable atypical melanocytes. One may infer from this observation that much of the melanoma risk was associated with the atypical melanocytes. Similar risk of cancer seemingly due to the development of atypical cells in precursor lesions is seen in breast and cervix neoplastic development (Ferenczy & Winkler, 1987; Dupont & Page, 1985; Page & Dupont, 1990). Thus, in heritable melanoma, the breast, and the cervix, atypical cells affording cancer risk are present in recognisable precursor lesions. Therefore, the development of atypical cells, randomly scattered within a normal tissue, should also be a risk factor for the development of cancer. Atypical melanocytes are seen in light-exposed skin of patients $>50$ years of age, who have type I or type II skin. These cells are a likely source of origin of cancer in direct tumour progression. Collectively, these randomly scattered atypical cells and the extensive atypia of Class IC lesions consitute the precursor states.

### A summary with a discussion of critical events in neoplasia

This paper proposes that the sequential events of tumour progression are similar in all neoplastic systems and these events are usually required for cancer to develop. Thus, tumour progression is a fundamental part of the 'intimate nature' of cancer (Nicolson, 1987). The following phenomena are encompassed under the concept of tumour progression as it relates to the development of cancer.

Known inductive mechanisms of human and experimental cancer produce a focal, clonal proliferation of cells that

**Table I** A COMPARISON OF THE LESIONS OF TUMOUR PROGRESSION IN NEOPLASIA
(Read down for tumour progression. Read across for prototypic lesions in different neoplastic systems)

| *Lesional class* | *Melanocytic neoplasia (human)* | *Hepatic neoplasia - rat* | *Keratinocytic neoplasia (mouse and human)* | *Colorectal neoplasia (human)* |
|---|---|---|---|---|
| *Class I* *Precursor lesions* | *Temporally restricted growth. Growth confined to the tissue compartment of origin* | | | |
| *1A. Initial lesion.* Clonal proliferation of structurally benign cells that disappears via differentiation. Progression to the subsequent steps of tumour progression is invariant in order but is not inexorable | *Ordinary mole* with regression via differentiation (melanocytic nevus) | *Microscopic islands followed by hepatocyte nodules* with regression via differentiation | *Squamous papilloma* with regression via differentiation | *Tubular adenoma (small adenoma)* with regression |
| *Class IB. Aberrant differentiation in intial lesion.* Focal growth of a subset of cells in a pattern different from the initial lesion. From this step forward differentiation is diminished, but not absent | *Abnormal pattern of melanocytic growth* in an ordinary mole | *Persistent nodules* with 'spontaneous' proliferation of a discrete subset of the total cell population of the nodule | *Squamous papilloma with failure of differentiation. Mitoses above the basal layer* | Intermediate adenoma |
| *Class IC. Dysplasia. Aberrant differentiation plus some cells with cytologic atypia* | *Melanocytic nevus with dysplasia* | *Persistent nodules* with spontaneous proliferation of a discrete subset of the total cell population of the nodule. Cell death and altered shut-off of the cell cycle. | *Squamous papilloma with atypia. Actinic keratosis* (human lesion) | *Tubulo-villous adenoma with severe atypia or large tubular adenoma with severe atypia* (late adenomas) |
| *Class II* *Intermediate lesions* | *Temporally unrestricted growth. Growth confined to tissue compartment of origin or is only microinvasive. Growth does not occur in mesenchyme of primary site* | | | |
| All cells of lesions are atypical and grow in contiguous array | 'Melanoma *in situ*'. Radial growth phase primary melanoma | Nodules within nodules | Squamous cell carcinoma *in situ*. Microinvasive carcinoma | Large tubulovillous adenoma with carcinoma *in situ*. Invasive carcinoma above the level of the muscularis mucosae |
| *Class III* *Primary cancer* | *Temporally unrestricted growth. Growth occurs in the mesenchyme of the primary site. Metastases may or may not occur* | | | |
| A nidus of cells in the primary tumour having most properties of cancer cells including the ability to involve three or more tissue compartments (metastasis). Properties related to metastasis are expressed in some but not all Class III lesions | Primary melanoma in the vertical growth phase. Thirty-seven per cent (37% – 121/328) exhibit metastatic competence. Nine years follow-up | Invasive hepatocellular cancer with potential for metastasis; a potential not always expressed | Invasive squamous cell carcinoma | Colorectal carcinoma extending into or through the muscularis mucosa |
| *Class IV* *Metastasis* | *The diverse patterns of metastatsis in primary stage I vertical growth phase melanoma* | | | |
| Lymph node metastases without subsequent metastases of any kind | Seven per cent (7% – 22/328) of vertical growth phase melanomas ... 18% (22/121) of tumours with metastasis. Nine years follow-up | | | |
| Blood vascular metastases with prior or concomitant lymph node metastases | Fourteen per cent (14% – 47/328) of vertical growth phase melanomas ... 39% (47/121) of tumours with metastasis. Nine years follow-up | | | |
| Blood vascular metastases without prior or concomitant lymph node metastases | Sixteen per cent (16% – 52/328) of vertical growth phase melanomas ... 43% (52/121) of tumours with metastasis. Nine years follow-up | | | |
| Metastases from metastases | | | | |

disappears via a programmed pathway of differentiation. This first response to a carcinogen has been termed the initial lesion. The form and behaviour of the initial lesion is the reference for the nature of aberrant form and behaviour in subsequent Class I lesions. The initial lesion is the manifestation of clonal adaptation as described by Farber and *progressive state selection* as described by Rubin (Farber, 1990; Rubin, 1990).

Different inductive agents produce the same kind of lesion. For example, chemical carcinogens, human papillomaviruses, and ultraviolet light all produce squamous papillomas as the initial lesion.

Failure of the initial lesion to disappear, commonly followed by an aberrant growth pattern within the initial lesion, is a cardinal step in tumour progression that may lead to overt cancer. Conversely, if the initial lesion follows its pathway of differentiation and disappearance cancer does not develop.

The various lesions with temporally restricted growth that may precede cancer are precursor lesions and are the Class I lesions of tumour progression.

Late Class I lesions (Class IC) contain many structurally atypical cells, cells similar in appearance to some cells appearing in invasive, primary cancers. These cells appear prior to biological cancer and may be the main source of the next lesional class.

The sequential lesions leading to the development of a primary cancer are the result of focal, not global, changes within a lesion.

Autonomous growth (temporally unrestricted growth) is the lesional property that distinguishes the later lesions of tumour progression from Class I lesions. This property is neither usually nor necessarily accompanied by capacity for metastasis. In fact, even the capacity for invasion is absent or quite limited. Lesions showing temporally unrestricted growth, but few other attributes of a completely evolved cancer, are Class II lesions.

Class III lesions are primary cancers: lesions showing temporally unrestricted growth, the ability to grow in the mesenchyme of the primary site and some ability to metastasise. Those Class III lesions with the ability to metastasise are fully evolved primary cancers.

Metastases are Class IV lesions. There are diverse metastatic pathways that may reflect subpopulations of cells in a primary cancer with properties required for completion of one metastatic pathway and not, necessarily, another.

The various classes of tumour progression lesions are clearly manifest in human, cutaneous melanocytic neoplasia. Keratinocytic, hepatic, and colonic neoplasia have been compared with melanocytic neoplasia and their development is similar.

Cancer, like time, presents no problem in definition until one is asked to define it. However, one cannot observe cancer and lesions related to its development over and over again without attempting to define it. In such an attempt one is forced, in the words of Garth Nicolson, '... into grandiose hypotheses that might explain the complex dynamic nature of cancers ...' (Nicolson, 1987). In the same article, Nicholson warns us, quoting Vriel, that 'no unitary concept can give a satisfactory explanation of the initimate nature of cancer'. I should leave the matter of the nature of cancer with Nicolson's warning, but already having indulged in 'grandiose hypotheses' and such things as cardinal properties, I must list the events that I regard as fundamental to the development of cancer and the development of metastasis. The concepts are not unitary, but pluralistic.

### *(1) The precursor states*

Cancers develop from precursor conditions that may result in large numbers of atypical cells. Farber has suggested that the initial lesions of neoplastic development (the hepatocyte nodule in hepatocarcinogenesis) are a physiologic response to cellular injury and are protective to the organism; the failure of the initial lesions to differentiate and disappear is the first indication that a pathway toward the development of cellular atypia has been evoked (Farber & Sarma, 1987). Aberrent growth and the apparance of atypical cells (dysplastic lesions) usually accompany the failure to differentiate: the pathway to cancer has begun. Yuspa has suggested that the process of carcinogenesis requires an alteration in the program of terminal differentiation in addition to aberrent growth control (Yuspa *et al.*, 1988). Evidence from the study of different neoplastic sysems suggests that this alteration of differentiation occurs as a flaw in the usual behaviour of initial lesions. Atypical cells similar to those of classical dysplastic lesions may develop without such lesions being manifest, possibly as a result of a concatenation of events occurring in single cells. The precursor conditions with their population of atypical cells are a necessary state for cancer to develop.

### *(2) Clonal transformation*

A lesion (an intermediate lesion) showing temporally unrestricted growth is the forerunner of cancer. It is posited that the intermediate lesions are the result of the clonal transformation of one of the atypical cells of the precursor state. The term transformation is borrowed from its *in vitro* usage and dose not imply tumourigenecity (Stanbridge *et al.*, 1982). These lesions may be regarded as the very beginnings of cancer and are usually termed '*in situ* malignancy'. They develop in one of many precursor sites, in one of many individuals affected by the precursor conditions of a neoplastic system. Most precursor lesions and conditions are end stage lesions with no future.

### *(3) Acquisition of metastatic competence and tumour cell heterogeneity by reciprocal interaction with the extracellular matrix*

Intermediate lesions show a tendency to persist and progress rather than to regress and become indolent. Growth and progression commonly lead to extension beyond the tissue compartment of origin and, in due course, to diminished synthesis of an intact basement membrane zone. With partial disappearance of the basement membrane zone, tumour cells interact with the nonbasement membrane extracellular matrix. It should be remembered, with regard to tumour cell-extracellular matrix interaction, that the tumour cells are abnormal. Consequently, the neoplastic parenchyma may be expected to invoke abnormalities in the extracellular matrix and the abnormal matrix to induce further abnormalities in the tumour cells; again and again, reciprocally. There could well be a conflation of genetic and nongenetic sources of information responsible for lesional behaviour in vertical growth phase melanomas (and its analogies) and in metastasis. Such reciprocal interaction is similar to that proposed and discussed by Mina Bissell (Bissell *et al.*, 1982). The cells of the tumour may then express an environmentally dependent (extracellular matrix) form of cellular inheritance. Harry Rubin considers such changes in tumours as one form of epigenesis, 'the acquisition of heritable characteristics without a covalent change in cellular DNA' (Rubin, 1990). Such heritable characteristics would not be stable, but dynamic due to a continuum of interation with the extracellular matrix (Rubin *et al.*, 1985). Vriel is doubtless correct in stating that no unitary concept can explain the 'intimate nature' of cancer. There is no oncogene, tumour suppressor gene, or chromosomal abnormality that is present routinely in cancer. In fact, some cancers have no demonstrable abnormalities of oncogenes or tumour suppressor genes and some are strictly diploid (Nicolson, 1987). Further, initial lesions, the response to carcinogens, are usually composed of diploid cells and the individual cells of initial lesions appear normal. Yet, and this is the main thrust of this paper, *all neoplasms develop in the same way, have the same general behavioural characteristics and, when malignant, they all interact with the extracellular matrix of the primary and the secondary sites.* They are unitary only in their developmental history and interaction with the extracellular matrix. Continued interaction with the extracellular matrix could be a form of natural selection. Such interaction with the matrix selects subsets of cells with heritable genetic and heritable epigenetic characteristics that use specific metastatic pathways. Late in the evolution of primary tumours, cells with metastatic competence may come to dominate the primary cancer (Kerbel *et al.*, 1988).

### *(4) Distant metastases as a continuum of reciprocal interaction with an extracellular matrix*

Metastases to distant organs are characterised by a continuation of reciprocal interaction with an extracellular matrix and adaptation to the specific matrix of that site. Except for lymph node metastases, a metastatic deposit is always in a mesenchyme. The last step in tumour progression is complete loss of influence of the microenvironment on the tumour (Nicolson, 1987). Such a last step is probably quite rare.

A list of the events and properties of a complete neoplastic system would be almost endless and similar in magnitude to the events of embryogenesis. There is not enough DNA nor abnormalities of DNA to explain neoplasia. That which is abnormal is not consistent from one neoplasm to the next. The magnitude of the problem is clearly expressed by R.C. Lewontin in his review of Edelman's book on Topobiology. I quote Lewontin, 'The problem is not one of dimension but of size. The nucleus of the cell of the fruit fly *Drosophila*, the favourite organism of the geneticists has enough DNA to specify the structure of about 5,000 different proteins and about 30 times that much DNA is available to provide spatial and temporal instructions about when the production of proteins by those genes should be turned on and turned

**Figure 4** The pattern of normal nevi in a 22 year old woman is shown. The appearance is characteristic of the initial lesions of melanocytic neoplasia (Class IA lesion). The nevi are small and relatively uniform in colour and outline. The lesions usually differentiate and disappear over the next three decades of life. **Figure 5** The clinical presentation of melanocytic nevi with aberrant differentiation and dysplasia in a 30 year old male patient who developed melanoma. The lesions are prototypic for Class IB and IC lesions of melanocytic neoplasia and manifest the beginning of tumour progression. Representative lesions are larger and more irregular in outline and colouration than normal nevi (Class IA). **Figures 6 and 7** The development of dysplastic nevi over a 6 year period in a male patient is shown. The patient's grandmother had melanoma and his father has dysplastic nevi. Figure 6 was taken at age 7 and Figure 7 at age 13. Class IB and IC lesions (aberrant differentiation and dysplasia) tend to slowly enlarge and then become indolent. Differentiation and disappearance is uncommon. **Figure 8** The photograph illustrates a malignant melanoma in the radial growth phase (Class IIB lesion) arising from a dysplastic nevus. The dysplastic nevus is the upper, irregular, tan-brown part of the lesion. The melanoma is the lower, black area. Class IIB lesions extend into the subjacent mesenchyme but do not metastasise. The cells in the dermis of radial growth phase melanomas usually show individual cells or small nests of cells surrounded by an intact basement membrane zone. **Figure 9** A malignant melanoma that has evolved from the radial to the vertical growth phase is shown. The picture is representative of a melanoma that has progressed to metastatic competence as manifested by the ability to grow in the mesenchyme of the primary site (Class III lesion). The upper centre and right portion of the photograph illustrate the radial growth phase. The centre shows an area of regression. The black nodule on the left is the vertifical growth phase partially surrounded by a remnant of a dysplastic nevus.

off. But this is simply too little, by many orders of magnitude, to tell every cell when it should divide, exactly where it should move next, and what cellular structures it should produce, over the entire developmental history of the fly. One needs to imagine an instruction manual that will tell every New Yorker when to wake up, where to go, and what to do, hour by hour, day by day, for the next century. There is just not enough DNA to go around' (Lewontin, 1989). To explain metazoan life or explain neoplasia in metazoan life.

Finally, it must be stated that tumour progression is not some engima hidden in the jargon of pathologists or molecular biologists. The lesions are not only visible, but their very appearance attests to their behaviour and, thus, to some aspect of their nature. The symmetry, differentiation and disappearance of initial lesions may be observed. The abnormalcy of aberrant differentiation is manifest as a large, asymmetrical nevus. The temporally unrestricted growth of intermediate lesions may be historically confirmed. Acquisition of competence for metastasis is reflected in changes in form of the primary cancer. All of the events of tumour progression are exceedingly complicated at the molecular level; at the level of the cell and the organism, however, tumour progression can be visualised and conceptualised. The accompanying illustrations permit one to visualise the sequence of lesional tumour progression events in human melanocytic neoplasia (Figures 4–9).

I wish to thank my associates in the Pigmented Lesion Study Group of the University of Pennsylvania, DuPont Guerry IV, David E. Elder, Allan Halpern, and Lynn Schucter for invaluable discussions concerning the nature of cancer and for specific suggestions for this manuscript.

Supported by grants from the National Institutes of Health, USA, CA-25298, CA-25874 and CA-16520.

### References

ARONSON, P.J., ITO, K., FUKAYA, T., HASHIMOTO, K. & MEHREGAN, A.H. (1988). Monoclonal antibody (AFH1) immunoreactive on morphologically abnormal basal melanocytes within dysplastic nevi, nevocellular nevus nests, and melanoma. *J. Invest. Dermatol.*, **90**, 452.

ASO, M., HASHIMOTI, K., ETO, H. & 4 others (1988). Expression of schwann cell characteristics in pigmented nevus. Immunohistochemical study using monoclonal antibody to Schwann cell associated antigen. *Cancer*, **62**, 938.

BERGMAN, W., RUITER, D.J., SCHEFFER, E., & VAN VLOTEN, W.A. (1988). Melanocytic atypia in dysplastic nevi. Immunohistochemical and cytophotometrical analysis. *Cancer*, **61**, 1660.

BISSELL, M.J., HALL, H.G. & PARRY, G. (1982). How does the extracellular matrix direct gene expression? *J. Theor. Biol.*, **99**, 31.

BURNS, F.J. (1989). Mouse skin papillomas as a stage in cancer. In *Skin Carcinogenesis: Mechanisms and Human Relevance*, Slaga, T., Klein-Szanto, A.J.P., Boutwell, R.K., Stevenson, D.E., Hugo, H.L. & D'Motto, B. (eds). Pp. 81–93. Alan R. Liss Inc: New York.

CLARK, W.H. (1991). Human cutaneous malignant melanoma as a model for cancer. *Cancer Met. Rev.*, **10**, 83.

CLARK, W.H., ELDER, D.E., GUERRY, D., EPSTEIN, M.N., GREENE, M.H. & VAN HORN, M. (1984). A study of tumor progression: the precursor lesions of superficial spreading and nodular melanoma. *Hum. Pathol.*, **15**, 1147. This paper also contains detailed and illustrated histologic descriptions of some of the phenomena discussed in the present paper.

CLARK, W.H., ELDER, D.E., GUERRY, D. & 5 others (1989). Model predicting survival in stage I melanoma based on tumor progression. *J. Natl Cancer Inst.*, **81**, 1893.

CLARK, W.H., ELDER, D.E., & GUERRY, D. (1990). Dysplastic nevi and malignant melanoma. In *Pathology of the Skin*, Farmer, E.R. & Hood, A.F. (eds). Pp. 684–756. Appleton & Lange: Norwalk.

DATA BASE OF THE PIGMENTED LESION STUDY GROUP, University of Pennsylvania, April 2, 1991.

DUPONT, W.D. & PAGE, D. (1985). Risk factors for breast cancer in women with proliferative breast disease. *N. Engl. J. Med.*, **312**, 146.

EDELMAN, G.M. (1989). Topobiology. *Sci. Am.*, **260**, 76.

ELDER, D.E., RODECK, U., THURIN, T. & 4 others (1989). Antigenic profile of tumor progression stages in human melanocytic nevi and melanomas. *Cancer Res.*, **49**, 5091.

FARBER, E.R. & SARMA, D.S.R. (1987). Biology of disease: hepatocarcinogenesis: a dynamic cellular perspective. *Lab. Invest.*, **56**, 4.

FARBER, E. (1990). Clonal adaptation during carcinogenesis. *Biochem. Pharmacol.*, **39**, 1837.

FEARON, E.R. & VOGELSTEIN, B. (1990). A genetic model for colorectal tumorigensis. *Cell*, **61**, 759.

FEINBERG, S.M., JAGELMAN, D.G., SARRE, R.G. & 5 others (1988). Spontaneous resolution of rectal polyps in patients with familial polyposis following abdominal colectomy and ileorectal anastomosis. *Dis. Col. Rectum*, **31**, 169.

FERENCZY, A. & WINKLER, B. (1987). Cervical intraepithelial neoplasia and condyloma. In *Blaustein's Pathology of the Female Genital Tract*. Kurman, R.J. (ed.). p. 188. Springer-Verlag: New York.

FOULDS, L. (1969). *Neoplastic Development*. Vol. 1. 41, Academic Press: New York.

GREENE, M.H., CLARK, W.H. Jr, TUCKER, M.A. & 7 others (1985*a*). Acquired precursors of cutaneous malignant melanoma: the familial dysplastic nevus syndrome. *N. Engl. J. Med.*, **312**, 91.

GREENE., M.H., CLARK, W.H., TUCKER, M.A., KRAEMER, K.H., ELDER, D.E. & FRASER, M.C. (1985*b*). High-risk malignant melanoma in melanoma-prone families with dysplastic nevi. *Ann. Intern. Med.*, **102**, 458.

HERLYN, M., CLARK, W.H., RODECK, U., MANCIANTI, M.L., JAMBROSIC, J. & KOPROWSKI, H. (1987). Biology of disease: biology of tumor progression in human melanocytes. *Lab. Invest.*, **56**, 461.

HOLZMANN, B., BROCKER, E.B., LEHMANN, J.M. & 4 others (1987). Tumor progression in human malignant melanoma: five stages defined by their antigenic phenotypes. *Int. J. Cancer*, **39**, 466.

HOWAT, A.J. & VARIEND, S. (1985). Lymphatic invasion in spitz nevi. *Am. J. Surg. Pathol.*, **9**, 125.

KERBEL, R.S., WAGHORNE, C., KORCZAK, B., LAGARDE, A. & BREITMAN, M.L. (1988). Clonal dominance of primary tumours by metastatic cells: genetic analysis and biological implications. *Cancer Surv.*, **7**, 597.

KLEIN-SZANTO, A.J.P. (1989). Morphological evaluation of the effects of carcinogens and promoters. In *Skin Carcinogenesis: Mechanisms and Human Relevance*, Slaga, T., Klein-Szanto, A.J.P., Boutwell, R.K., Stevenson, D.E., Hugo, H.L. & D'Motto, B. (eds), Pp. 45–62. Alan R. Liss: New York.

LEA, P.J. & PAWLOWSKI, A. (1986). Human melanocytic nevi. *Acta Dermato-Venereol.*, **127** (Suppl), 1.

LEWONTIN, R.C. (1989). The science of metamorphoses. *New York Review of Books*, April 27, p. 18.

LIOTTA, L.A., GUIRGUIS, R. & STRACKE, M. (1987). Biology of melanoma invasion and metastasis. *Pigment Cell Res.*, **1**, 5.

MASRI, G.D., CLARK, W.H., GUERRY, D., HALPERN, A., THOMPSON, C.J. & ELDER, D.E. (1990). Screening and surveillance of patients at high risk for malignant melanoma result in detection of earlier disease. *J. Am. Acad. Dermatol.*, **22**, 1042.

MORIKAWA, K., WALKER, S.M., NAKAJIMA, M., PATHAK, S., JESSUP, J.M. & FIDLER, I.J. (1988). Influence of organ environment on the growth, selection and metastasis of human colon carcinoma cells in nude mice. *Cancer Res.*, **48**, 6863.

NEWTON, J.A., CAMPLEJOHN, R.S. & MCGIBBON, D.H. (1988). The flow cytometry of melanocytic skin lesions. *Br. J. Cancer*, **58**, 606.

NICOLSON, G.L. (1987). Tumor cell instability, diversification and progression to the metastatic phenotype: from oncogene to oncofetal expression. *Cancer Res.*, **47**, 1473.

NOWELL, P.C. (1989). Chromosomal and molecular clues to tumor progression. *Semin. Oncol.*, **16**, 116.

PAGE, D.L. & DUPONT, W.D. (1990). Anatomic markers of human premalignancy and risk of breast cancer. *Cancer*, **66**, 1326.

PARMITER, A.H. & NOWELL, P.C. (1988). The cytogenetics of human malignant melanoma and premalignant lesions. In *Malignant Melanoma: Biology, Diagnosis and Therapy*. Nathanson, L. (ed.), p. 47. Kluwer Academic Publishers: Boston.

PARMITER, A.H., BALABAN, G., CLARK, W.H. & NOWELL, P.C. (1988). Possible involvement of the chromosome region 10q24→26 in early stages of melanocytic neoplasia. *Cancer Genet. Cytogenet.*, **30**, 313.

PLAZ, C.E. (1978). The staging and pathology of colonic carcinoma and adenomas. In *Carcinoma of the Colon and Rectum*, Enker, W.E. (ed.), Pp. 21-48. Year Book Medical Publishers Inc: Chicago.

PREHEN, R.T. (1990). Lymphoid dependency and cutaneous melanoma. *J. Natl Cancer Inst.*, **82**, 626.

REED, R.J. (1988). Minimal Deviation Melanoma. In *Pathobiology and Recognition of Malignant Melanoma*, Mihm, M.C., Murphy, G.F. & Kaufman, N. (eds), Pp. 110-152. Williams & Wilkins: Baltimore.

RHODES, A.R., SEKI, Y., FITZPATRICK, T.B. & STERN, R.S. (1988). Melanosomal alterations in dysplastic nevi: a qualitative ultrastructural investigation. *Cancer*, **61**, 358.

RUBIN, H. (1990). On the nature of enduring modifications induced in cells and organisms. *Am. J. Physiol.*, **258**, 19.

RUBIN, H. (1985). Cancer as a dynamic developmental disorder. *Cancer Res.*, **45**, 2935.

SAGEBIEL, R.W. (1991). Personal communication.

SCHMOECKEL, C., STOLZ, W., SAKAI, L., BURGESON, R.E., TIMPL, R. & KREIG, T. (1989). Structure of basement membranes in malignant melanoma and nevocytic nevi. *J. Invest. Dermatol.*, **92**, 663.

SMITH, K.J., BARETT, T.L., SKELTON, H.G., LUPTON, G.P. & GRAHAM, J.H. (1989). Spindle cell and epitheloid cell nevi with atypia and metastasis (malignant spitz tumor). *Am. J. Surg. Pathol.*, **13**, 931.

STANBRIDGE, E.J, DER, C.J., DOERSEN, C.J. & 4 others (1982). Human cell hybrids: analysis of transformation and tumorigenicity. *Science*, **215**, 252.

WILLIAMS, G.M. (1980). The pathogenesis of rat liver cancer caused by chemical carcinogens. *Biochem. Biophys. Acta*, **605**, 167.

YAAR, M., WOODLEY, D.T. & GILCHRIST, B.A. (1988). Human nevocellular nevus cells are surrounded by basement membrane components. Immunohistologic studies of human nevus cells and melanocytes *in vivo* and *in vitro*. *Lab. Invest.*, **58**, 157.

YUSPA, S., KILKENNY, A. & ROOP, D.R. (1988). Aberrant differentiation in mouse skin carcinogenesis. *IARC Sci. Publ.*, **92**, 3.

ZETTER, B.R. (1990). The cellular basis of site-specific tumor metastasis. *N. Engl. J. Med.*, **322**, 605.